# Intermittent Hypoxia Associated with Sleep Apnea Disrupts Microvascular Hemodynamics and Oxygen Delivery

**DOI:** 10.1038/s42003-026-10027-z

**Published:** 2026-04-11

**Authors:** Carlos Munoz, Jacinda Martinez, Daniela Lucas, Zhixuan Song, Sophia Lin, Pedro Cabrales

**Affiliations:** Department of Bioengineering, University of California, San Diego, La Jolla, CA USA

**Keywords:** Vascular diseases, Molecular medicine

## Abstract

Obstructive sleep apnea (OSA) is characterized by recurrent episodes of intermittent hypoxia (IH). In this study, we investigated how cyclic IH (21%/10% O₂) acutely alters systemic hemodynamics and microvascular vascular tone, hemodynamics, and oxygen transport compared to the control (21%/21% O₂). Using a dorsal window chamber model on unanesthetized Golden Syrian hamsters, we applied 30-second oxygen cycling for 60 minutes. Microvascular oxygen saturation was continuously monitored using hyperspectral imaging (HSI), and vessel diameter, red blood cell velocity, functional capillary density (FCD), and vascular resistance were quantified through intravital microscopy. Animals exposed to IH exhibited significant reductions in mean arterial pressure and arterial oxygen saturation, along with increased heart rate and blood lactate. Microvascular SO₂ declined rapidly in the microcirculation and stabilized after 8 mins. Arteriolar blood flow decreased, and FCD was significantly reduced relative to both baseline and control. Despite a decrease in vascular resistance, vasodilatory compensation was insufficient and resulted in decreased oxygen delivery (DO₂) and oxygen extraction (VO₂) to tissues. The oxygen extraction ratio increased, suggesting a limited capacity to offset hypoxic stress. These findings demonstrate that cyclic IH significantly disrupts peripheral microcirculatory flow and oxygenation, highlighting the relevance of IH models in assessing oxygen transport during OSA.

## Abstract (web summary)

Using hyperspectral imaging and intravital microscopy, this study reveals how cyclic hypoxia impairs microvascular perfusion and tissue oxygenation.

## Introduction

Sleep disordered breathing includes brief, often cyclical interruptions in breathing rhythm (apneas) or temporary or sustained reductions in breath amplitude (hypopneas), which can cause significant arterial hypoxemia and hypercapnia^1^. These events occur specifically during sleep and may involve: (1) a compromised, sometimes fully closed extrathoracic upper airway (“obstructive” event); (2) a significant reduction or cessation of brainstem respiratory motor output (“central” event); and (3) a combination of both central and obstructive events^2^. Consequently, individuals undergo recurrent cycles of intermittent hypoxia (IH), and this IH trigger physiological changes in cerebral oxygenation^3^, endothelial dysfunction^4^, platelet activation^5^, inflammation and irregular cytokine profiles^6^. Furthermore, the field of sleep disorder research has recently gained momentum; however, much remains unknown, particularly regarding the effect of apnea in the microcirculation, specifically hemodynamics and tissue oxygenation.

In recent years, the microcirculation has attracted growing interest not only from basic scientists but also from clinicians and translational researchers^7,8^. Clinically, impaired microcirculatory function has been convincingly shown to predict outcomes in critically ill patients^9^, and it is increasingly recognized as a potential therapeutic target in this population^10,11^. The development of modern diagnostic tools, including non-invasive methods to quantify microvascular perfusion, has enabled more individualized treatment strategies aimed at optimizing organ perfusion and oxygenation. A substantial body of evidence underscores the central role of the smallest blood vessels in key physiological processes, including inflammation, hyperviscosity, cell–cell interactions, endothelial function, tissue edema, hemodynamic regulation, oxygenation, and inflammatory cell interactions with cellular and soluble mediators^7^. Perhaps most importantly, microvascular perfusion is essential for the exchange of gases, nutrients, and the removal of cellular waste products. One of the most significant pathological changes across a broad range of disorders occurs when tissues or cells are deprived of oxygen, i.e., under hypoxic conditions^12^. For clinicians, the priority is to restore oxygenation and prevent irreversible tissue damage; for basic scientists, the focus lies in unraveling the pathophysiological mechanisms of hypoxia in search of regulatory master switches and therapeutic opportunities.

In this study, we utilize various tools to observe microvascular physiological responses in the dorsal skin fold of conscious Golden Syrian hamsters during IH. We coupled in vivo measurements of microvascular hemodynamics with noninvasive imaging of microvascular oxygen saturation to create a comprehensive profile of microvascular oxygenation during 30-s intervals of oxygen cycling between 21% and 10% oxygen levels. We hypothesize that, in response to this hypoxic burden, systemic compensatory adjustments and microvascular adaptations may be insufficient to preserve effective oxygen delivery, resulting in reduced tissue oxygen availability and altered hemodynamics.

## Method

### Animal preparation

**Fig. 1:**
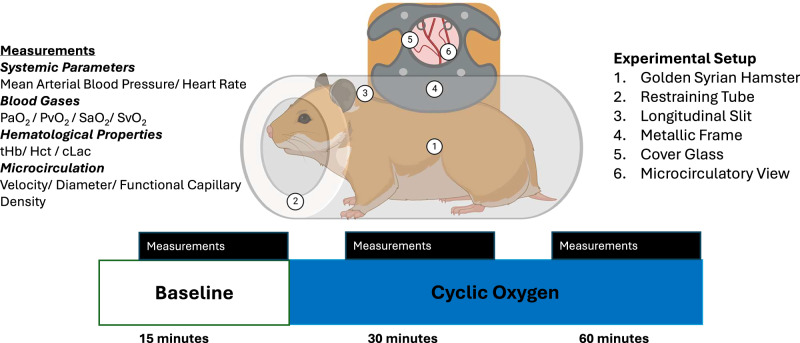
Schematic of the experimental setup and timeline. Evaluating systemic and microcirculatory responses to cyclic oxygen exposure in a Golden Syrian hamster dorsal window chamber model. The hamster (1) was placed in a restraining tube (2) with a longitudinal slit (3) to allow access to the dorsal skinfold chamber, which was secured with a metallic frame (4) and cover glass (5) for real-time microcirculatory imaging (6). Measurements were taken at baseline and at 30 and 60 min during the exposure phase. Parameters assessed included systemic variables (mean arterial pressure, heart rate), blood gases (pO₂, pCO₂, sO₂), hematological markers (total hemoglobin, hematocrit, lactate), and microcirculatory metrics (red blood cell velocity, diameter, and functional capillary density). In the experimental group, oxygen was cycled between 21% and 10% every 30 s, while the control group received 21% continuously (cycled 21%/21%). In both groups, air was delivered directly into the restraining tube at a constant flow rate of 1 L/min.

Male Golden Syrian hamsters (Charles River Laboratories, Boston, MA; age 3–4 weeks; body weight 55–65 g) were used in this study. Animals were randomly assigned to either an intermittent hypoxia (IH; *n* = 8) or control (*n* = 8) group. Only male animals were used to minimize biological variability associated with estrous-cycle–dependent hormonal fluctuations known to affect vascular tone and microcirculatory regulation. Sex as a biological variable was not assessed, and conclusions are therefore limited to male physiology. Animal handling and care followed the NIH Guide for the Care and Use of Laboratory Animals. The experimental protocol was approved by the local animal care committee. The hamster window chamber model is widely used for microvascular studies in the unanesthetized state, and the complete surgical technique is described in detail elsewhere^13,14^. Briefly, to minimize restraint-related stress during conscious measurements, the animals were acclimatized to the restraining tube prior to preparation. Upon arrival, the same tube used during imaging was placed in the home cage for at least three days to allow voluntary exploration and habituation; this approach leverages the species’ natural preference for enclosed, burrow-like environments. The preparation begins with anesthetizing the animal and creating a dorsal skinfold by extending a 2.5-cm section of hamster skin from the back. A circular portion of skin and underlying tissue, measuring 1.5 cm in diameter, is completely removed from one side of the fold, thereby exposing the underlying tissue on the opposite side. The overlapping fascia in the exposed area is carefully separated and retracted to allow access to the tissue beneath. Animals were allowed to recover for 1 day, after which the window chamber was examined under a microscope for signs of edema, bleeding, or abnormal neovascularization. Animals were then re-anesthetized, and catheters were implanted in the carotid artery and jugular vein using heparinized saline (30 IU/mL). The catheters were tunneled subcutaneously, exteriorized at the dorsal aspect of the neck, and securely affixed to the window chamber frame. Two days after catheter implantation, the microvasculature was re-examined, and only animals whose window chambers showed no evidence of low perfusion, inflammation, or edema were included in the study. The animal preparation procedure is illustrated in Fig. 1.

### Inclusion criteria

Animals were suitable for the experiments if (1) systemic variables were within a normal range, namely, heart rate (HR) > 340 beat/min, mean arterial blood pressure (MAP) > 80 mmHg, systemic hematocrit (Hct) > 45%, and arterial oxygen partial pressure (PaO_2_) > 50 mmHg; and 2] microscopic examination of the tissue in the chamber observed under a 650× magnification did not reveal signs of edema or bleeding. Inclusion criteria for arterial pO₂ (≥50 mmHg) were selected to reflect hamster-specific systemic physiology rather than rodent models with higher baseline pO₂. As a fossorial species, hamsters exhibit lower arterial pO₂ due to adaptation to subterranean environments; however, microvascular pO₂ distribution in the dorsal window chamber model is comparable to that observed in mice and rats^15,16^.

### Simulated intermittent cyclical hypoxia (apneas)

Unanesthetized animals were placed in a small (5 cm diameter and 15 cm length) restraining tube with a longitudinal slit, allowing the dorsal window chamber to extend outside for observation. The restraining tube was supplied with oxygen at a controlled flow rate of 1 L/min through a motorized gas-switching valve system alternating gases every 30 seconds. In the intermittent hypoxia (IH) group (*n* = 8), oxygen alternated between 21% (normoxic) and 10% (hypoxic) concentrations, while in the control (Control) group (*n* = 8), the gas alternated between 21% and 21% O_2_ from two different sources to ensure an identical gas-switching setup without changes in oxygen concentration. Given the small residual gas volume within the restraining tube (~30 mL) and a delivery flow rate of 1 L/min, complete gas exchange occurs within seconds, ensuring rapid onset of hypoxic exposure during each cycle. This design allowed the animal to breathe freely while enabling real-time monitoring of systemic and microvascular physiological responses to dynamic changes in O_2_ concentration.

### Systemic variables and blood chemistry

Physiological monitoring and blood analysis were performed during entire study. The arterial catheter was connected to a pressure transducer and recording system (MP150, Biopac, Santa Barbara, CA), and blood pressure signals was recorded, along with MAP, and HR. The Hct was measured from centrifuged arterial blood samples taken in heparinized capillary tubes. Hb content was determined spectrophotometrically from a single drop of blood (B-Hemoglobin, Hemocue, Stockholm, Sweden). Arterial blood was collected in heparinized glass capillaries (50 μL) and immediately analyzed for pO_2_, pCO_2_, pH, electrolytes, lactate, and total Hb (tHb) content (ABL90; Radiometer America, Brea, CA). The results are found in Tables 1 and 2.

**Table 1:** Systemic and blood gas parameters measured at baseline, 30 min, and 60 min during exposure to either control (21%/21% O₂) or intermittent hypoxia (21%/10% O₂) conditions

	Baseline		30 min		60 min	
	Control	Intermittent Hypoxia	Control	Intermittent Hypoxia	Control	Intermittent Hypoxia
**Hct (%)**	46 ± 2	46 ± 2	45 ± 2	44 ± 2	45 ± 2	44 ± 2
tHb (g/dL)	14.7 ± 0.7	14.7 ± 0.6	14.1 ± 1.1	14.1 ± 0.9	14.1 ± 1.3 †	14.1 ± 1.1
MAP (mmHg)	122.3 ± 7.6	116.9 ± 6.5	113.3 ± 9.2	100.6 ± 11.3 †	113.0 ± 12.6	94.2 ± 16.9 †
HR (bpm)	449.9 ± 35.7	428.11 ± 32.5	428.7 ± 45.8	482.4 ± 35.2	442.3 ± 36.1	441.9 ± 43.8
PaO_2_ (mmHg)	62.3 ± 9.1	67.9 ± 10.5	55.7 ± 8.2	34.1 ± 9.6 †	61.2 ± 9.3	31.9 ± 5.2 †
PaCO_2_ (mmHg)	59.2 ± 6.9	55.5 ± 5.4	56.4 ± 5.5	42.9 ± 5.0 †	56.5 ± 5.4	44.6 ± 4.4 †
Arterial pH	7.34 ± 0.1	7.38 ± 0.05	7.36 ± 0.03	7.44 ± 0.05	7.35 ± 0.02	7.43 ± 0.06
SO_2_ (%)	89.5 ± 6.5	92.5 ± 2.6	86.5 ± 5.0	60.6 ± 3.6 †	90.6 ± 3.7	60.1 ± 9.8 †
† to baseline						

Oxygen was cycled every 30 s and delivered directly into the restraining tube at a flow rate of 1 L/min. Each group consists of 8 individual animals (*n* = 8 per group). Values are expressed as mean ± SD with statistical analyses performed using mean values.

† indicates significant difference from baseline (*P* < 0.05).

**Table 2:** Electrolyte and lactate concentrations measured at baseline, 30 min, and 60 min during exposure to control (21%/21% O₂) or intermittent hypoxia (21%/10% O₂) conditions

	Baseline		30 min		60 min	
	Control	Intermittent hypoxia	Control	Intermittent hypoxia	Control	Intermittent hypoxia
**K**^**+**^ **(mM)**	5.1 ± 0.7	5.1 ± 0.4	5.0 ± 0.7	4.7 ± 0.4 †	5.0 ± 0.6	4.4 ± 0.5 †
Na^+^ (mM)	138.9 ± 1.6	136.8 ± 2.5	138.6 ± 1.1	136.0 ± 2.5	138.3 ± 0.5	136.6 ± 2.1
Ca^2+^ (mM)	1.3 ± 0.1	1.3 ± 0.1	1.3 ± 0.1	1.2 ± 0.1	1.2 ± 0.1	1.2 ± 0.1
Cl^-^ (mM)	99.1 ± 2.3	95.9 ± 2.6	98.6 ± 1.9	96.6 ± 2.1	99.6 ± 3.3	98.0 ± 3.7
Lac (mM)	1.3 ± 1.0	1.1 ± 0.5	1.1 ± 0.5	2.6 ± 1.3	0.8 ± 0.3	1.9 ± 0.8
† to baseline						

Oxygen was cycled every 30 s and delivered directly into the restraining tube at a constant flow rate of 1 L/min. Each group consists of 8 individual animals (*n* = 8 per group). Values are expressed as mean ± SD with statistical analyses performed using mean values.

† indicates significant difference from baseline (*P* < 0.05).

Baseline measurements were obtained under steady normal conditions (21% O₂) following a 20-min stabilization period and prior to initiation of gas cycling. Intermittent hypoxia was then applied (21%/10% O₂; 30 s per phase), and measurements at 30 and 60 min were acquired over multi-minute windows during continuous cycling, yielding values integrated across multiple hypoxic–normoxic transitions.

### Microvascular experimental setup

On the day of imaging, the unanesthetized animals were placed in a small restraining tube then fixed to the microscopic stage of a custom transillumination intravital microscope (BX51WI, Olympus, New Hyde Park, NY). The animals were given 20 min to adjust to the change in the tube environment before measurements were made. The tissue images were projected onto a charge-coupled device camera (COHU 4815, San Deigo. CA), and connected to a videocassette recorder and a monitor. Measurements were carried out using a 40× (LUMPFL-WIR, NA 0.8, Olympus. Japan) water immersion objective. The same sites of study were followed throughout the entire experiment so comparisons could be made directly to baseline levels.

Similar to how the systemic varabiles and blood gases were collected, baseline measurements were obtained under steady normal conditions (21% O₂) following a 20-min stabilization period and prior to initiation of gas cycling. Intermittent hypoxia was then applied (21%/10% O₂; 30 s per phase), and measurements at 30 and 60 min were acquired over multi-minute windows during continuous cycling, yielding values integrated across multiple hypoxic–normoxic transitions.

### Microhemodynamics

Arteriolar and venular blood flow velocities were measured online by using the photodiode cross-correlation method^17^ (Photo Diode/Velocity Tracker Model 102B, Vista Electronics, San Diego, CA). Briefly, the tissue image is projected on two photodiodes placed between the charge-coupled device camera and the inverted microscope allowing for the light from the bottom of the microscope to radiate through the tissue and onto the photodiode. The measured centerline velocity (*V*) was corrected according to vessel size to obtain the mean RBC velocity^18^. A video image-shearing method was used to measure vessel diameter (*D*)^19^. The precision and consistency of measurements obtained through image shearing with the television camera were directly influenced by the camera’s linearity and the operator’s level of training. It was observed that using an RCA PK20 with a Vidicon image tube could achieve an accuracy of approximately 0.2% of the video image width. Therefore, when a microscopic blood vessel with a diameter of 10 µm is magnified so that its diameter occupies half of the video width, the measurement accuracy is around 0.1 µm. Blood flow (*Q*) was calculated from the values measured as *Q* = *π* × *V* × (*D*/2)^2^. The velocity and diameter of each individual vessel were recorded independently within the first 10 min of starting the measurement to minimize variability in the microcirculation due to time.

Before baseline measurements, venules and arterioles were mapped separately, and each blood vessel selected was evaluated for diameter and blood flow. Arterioles and venules were distinguished based on flow direction: vessels where flow branched directly from daughter to collecting vessels were identified as venules, while those with flow moving from feeding to daughter branches were classified as arterioles. The data was divided into three groups: arterioles with diameter below 60 µm (<60 µm), arterioles with a diameter above 60 µm (>60 µm), and venules with a diameter between 20 and 80 µm. These groups were categorized based on their expected impact on vascular resistance. Vessels larger than 60 µm are identified as feeding arterioles and exhibit limited dilation or constriction (<20%). Conversely, vessels smaller than 60 µm include arcading, transverse, and terminal arterioles, capable of dilation or constriction greater than 20%. Venules are known for their compliance in response to volume and pressure changes influenced by arteriole activity.

Vascular resistance (*R*) for each vessel was calculated using the formula *R* = *P*/*Q* where *P* was approximated using mean arterial pressure (MAP), and *Q* was the measured microvascular blood flow. Because microvascular pressure was not measured directly, MAP was used as a surrogate for upstream perfusion pressure, and vascular resistance was calculated as MAP/flow and reported as a baseline-normalized, unitless ratio.

### Functional capillary density

Functional capillaries, defined as capillary segments that showed RBC transit of at least one RBC within a 45-s period across approximately 10 consecutive microscopic fields (0.05 mm² each), were evaluated, covering a total area of 0.5 mm² of the total window chamber. Each field contained between five and ten capillary segments with RBC flow. A capillary was defined as a vessel through which a single RBC flowed in a linear fashion. FCD (cm^−1^), i.e., the total length of RBC perfused capillaries divided by the area of the microscopic field of view, was evaluated by measuring and adding the length of capillaries that exhibited RBC transit in the field of view. The relative change in FCD from baseline levels after each intervention is indicative of the extent of capillary perfusion^20,21^.

### Microvascular oxygen saturation and delivery

Unanesthetized animals instrumented with the dorsal window chamber were imaged using a Pika L hyperspectral imaging system (Resonon Inc., Bozeman, MT) to collect hyperspectral images (HSI) of the microcirculation. The methods for analyzing these images have been previously described^22^. Images were taken at baseline, every minute for the first 10 min, and then every 3 min for the next 50 min, for a total experiment duration of 60 min. HSI was used to quantify intravascular oxygen saturation (SO₂) in both arterioles and venules across the entire microvascular network.

Oxygen delivery (DO₂) was calculated as the product of arteriolar blood flow (*Q*, in nL/s) and arteriolar oxygen content, derived from Hb SO_2_ and Hb concentration. Oxygen extraction (VO₂) was calculated as the product of the difference in oxygen content between arterioles and venules and the measured venular blood flow. The oxygen extraction ratio (OER), defined as the percentage of O_2_ used, was computed as the ratio between VO₂ and DO₂ (VO_2/_DO_2_) at each time point. Together, these parameters provided a dynamic assessment of microvascular O₂ transport under cyclic hypoxic and normoxic conditions.

### Statistical analysis

Results are presented as box-and-whisker plots to illustrate data distribution, variability, and outliers, as well as line plots depicting mean ± standard deviation to highlight temporal trends across experimental conditions, and tabulated data is presented as mean ± standard deviation. Box-and-whisker plots display the median (center line), interquartile range (box; 25th–75th percentiles), and 5th–95th percentiles (whiskers). Although medians are emphasized in box-and-whisker plots for visual clarity, statistical comparisons were performed using mean values, consistent with the parametric and nonparametric tests applied.

Within-group time-dependent effects were analyzed using repeated-measures analysis of variance (ANOVA) or the Kruskal–Wallis test when data did not meet normality assumptions. When appropriate, post hoc comparisons were conducted using Dunn’s multiple-comparison test. Comparisons between experimental groups were performed using two-way ANOVA, considering treatment and time as independent variables, followed by Bonferroni post hoc tests.

Microhemodynamic parameters are presented as ratios relative to baseline values. A ratio of 1.0 indicates no change from baseline, whereas values below or above 1.0 represent proportional decreases or increases, respectively (e.g., 1.5 indicates a 50% increase from baseline). The same blood vessels and capillary fields were tracked throughout the study, enabling direct comparisons to baseline measurements and improving statistical robustness despite small sample sizes. Oxygen saturation is reported as a fraction of the total.

All statistical analyses were performed using GraphPad Prism 9 (GraphPad Software, San Diego, CA). Differences were considered statistically significant at *p* < 0.05. Sample size estimation was based on ANOVA power calculations (*α* = 0.05, power = 0.9), resulting in a minimum acceptable sample size of 15 microvessels per group.

### Reporting summary

Further information on research design is available in the Nature Portfolio Reporting Summary linked to this article.

## Results

### Systemic hemodynamic and blood gas responses to intermittent hypoxia

**Fig. 2:**
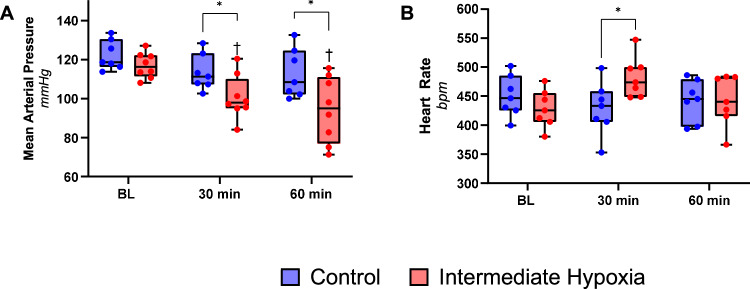
Systemic hemodynamic parameter. Mean arterial pressure (**A**) and heart rate (**B**) measured at baseline (BL), 30 min, and 60 min during exposure to control (21%/21% O₂) or intermittent hypoxia (21%/10% O₂) cycling conditions. Oxygen was cycled every 30 s and delivered directly into the restraining tube at 1 L/min. Data are displayed as box-and-whisker plots, where the center line represents the median, the box denotes the interquartile range (25th–75th percentiles), and the whiskers indicate the 5th–95th percentiles. Intermittent hypoxia resulted in a significant reduction in MAP at 30 and 60 min compared to both baseline and control. Heart rate was significantly elevated at 30 min in the intermittent hypoxia group compared to control. Each group consists of 8 individual animals (*n* = 8 per group). Statistical analyses was performed using mean values. † indicates significant difference from baseline (*P* < 0.05).

Exposure to cyclic intermittent hypoxia (21%/10% O₂) led to significant systemic changes relative to both baseline and normoxic control conditions. The MAP decreased progressively over time in the IH group, with statistically significant reductions observed at both 30 and 60 min compared to baseline and to control animals at the same time points, as shown in Fig. 2A. This suggests a loss of vascular tone or impaired pressure regulation under hypoxic cycling. Conversely, heart rate (HR) increased significantly in the IH group at 30 min compared to control, likely reflecting sympathetic activation (Fig. 2B). After 60 min, HR trended downward, potentially indicating autonomic fatigue. Control animals maintained stable MAP and HR throughout the duration of exposure.

Blood gas analysis (Table 1) confirmed systemic hypoxemia in the IH group, with reduced arterial pO₂ and SaO₂ compared to baseline, particularly after 60 min. Venous SO_2_ levels were also markedly lower, reflecting both reduced DO_2_ and increased O_2_ extraction. In contrast, the control group maintained consistent oxygenation levels during the entire observation period. Lactate levels increased in the IH group after 30 min (Table 2), indicating a shift toward anaerobic metabolism due to insufficient tissue oxygenation, but trended downwards after 60 min. Electrolyte measurements showed a significant reduction in plasma potassium levels, which may reflect cellular compensation mechanisms or ionic imbalances associated with stress and hypoxia. The control group did not show any significant changes in lactate or electrolytes.

### Microvascular hemodynamics

**Fig. 3:**
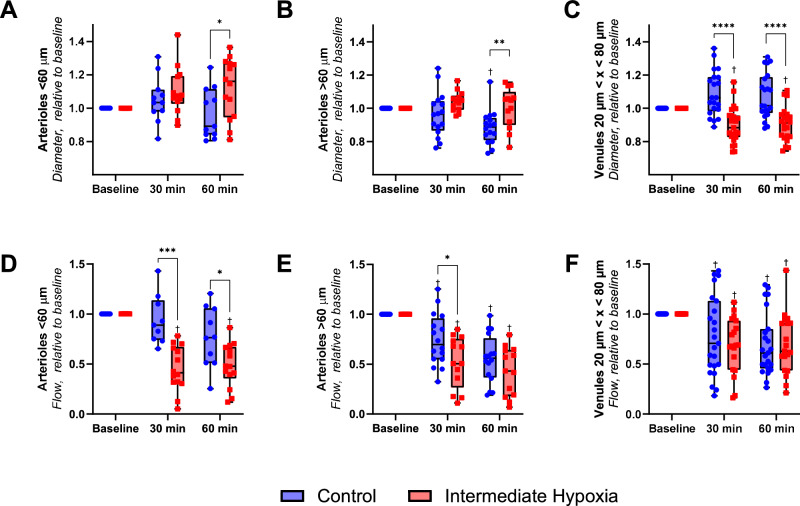
Microvascular hemodynamics. Relative changes in microvascular diameter (**A**–**C**) and blood flow (**D**–**F**) in arterioles and venules during exposure to control (21%/21% O₂) or intermittent hypoxia (21%/10% O₂) cycling conditions. Measurements were obtained at baseline, 30 min, and 60 min, with oxygen cycled every 30 s at 1 L/min. Arteriolar diameter (<60 μm and >60 μm) and venular diameter (20–80 μm) are shown in panels (**A**–**C**), and corresponding blood flow values are shown in panels (**D**–**F**); all data are normalized to baseline. Baseline values (mean ± SD) for controls were: arteriolar diameter 42 ± 16 μm (<60 μm) and 80 ± 12 μm (>60 μm), blood flow 2.5 ± 1.8 and 12.9 ± 8.1 nL/s, respectively, and venular diameter 58 ± 12 μm with flow 1.4 ± 1.2 nL/s. For the intermittent hypoxia group, baseline arteriolar diameters were 39 ± 10 μm (<60 μm) and 73 ± 10 μm (>60 μm), with blood flows of 2.1 ± 1.3 and 10.9 ± 5.6 nL/s, respectively; venular diameter was 57 ± 15 μm with flow 1.3 ± 1.6 nL/s. Data are shown as box-and-whisker plots (median, interquartile range, 5th–95th. Intermittent hypoxia resulted in increased arteriolar diameter, reduced venular diameter, and a decrease in blood flow across both arterioles and venules compared with baseline. Each group includes 16 independent vessels obtained from different animals (*n* = 4 per group). † indicates significant difference from baseline (*P* < 0.05), and * (*P* < 0.05), ** (*P* < 0.01), *** (*P* < 0.001), **** (*P* < 0.0001) indicate significant differences from control.

Cyclic intermittent hypoxia had profound effects on microvascular diameter and blood flow effects (Fig. 3). Small arterioles (<60 µm in diameter) demonstrated significant lack of vasoconstriction after 60 min in the intermittent hypoxia (IH) group compared with the control group. Similarly, large arterioles (>60 µm) also exhibited significant lack of vasoconstriction under IH conditions at 60 min; however, the magnitude of the difference relative to control was more pronounced in large arterioles than in small arterioles. Conversely, small venules with diameters between 20 and 80 µm demonstrate significant vasoconstriction at 30 and 60 min in the IH group compared to baseline and the control group.

Corresponding to these geometrical changes, blood flow decreased significantly in both arterioles and venules in the IH group (Fig. 3D–F). Small arterioles showed the greatest reduction in blood flow, aligning with their role in distributing capillary perfusion. Larger arterioles and venules also exhibited diminished blood flow, suggesting that intermittent hypoxia impairs not only downstream delivery but also upstream hemodynamic support.

### Changes in microvascular resistance

**Fig. 4:**
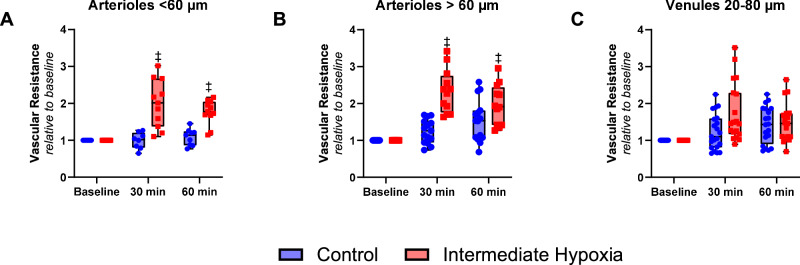
Vascular resistance. Vascular resistance in **A** small arterioles (<60 μm), **B** large arterioles (>60 μm), and **C** venules (20–80 μm) at baseline, 30 min, and 60 min under control (21%/21% O₂) and intermittent hypoxia (21%/10% O₂) conditions. Vascular resistance is reported as a relative, unitless value normalized to baseline in order to emphasize changes in vascular tone while minimizing inter-vessel variability in absolute pressure–flow relationships. In both arteriole size groups, vascular resistance decreased over time, indicating vasodilation. This reduction was more pronounced in the intermittent hypoxia group, particularly in small arterioles, suggesting an early compensatory response to accumulated hypoxia. Venular resistance also declined slightly in both groups but showed less overall variability. Data are displayed as box-and-whisker plots, where the center line represents the median, the box denotes the interquartile range (25th–75th percentiles), and the whiskers indicate the 5th–95th percentiles. Statistical analyses was performed using mean values of each individual vessel (*n* = 16 independent vessels per group). † indicates a significant difference from baseline in the control group; ‡ indicates a significant difference from baseline in the intermittent hypoxia group.

With a reduction in MAP and blood flow, vascular resistance increased over time in both the IH and control groups, across small arterioles (<60 μm), large arterioles (>60 μm), and venules (20–80 μm) (Fig. 4). As expected, the increase in vascular resistance was more pronounced in the IH group, with the greatest deviation from baseline in larger arterioles followed by the small arterioles and finally the venules. In contrast, the control group exhibited a mild increase in resistance over time.

### Capillary perfusion and functional capillary density

**Fig. 5:**
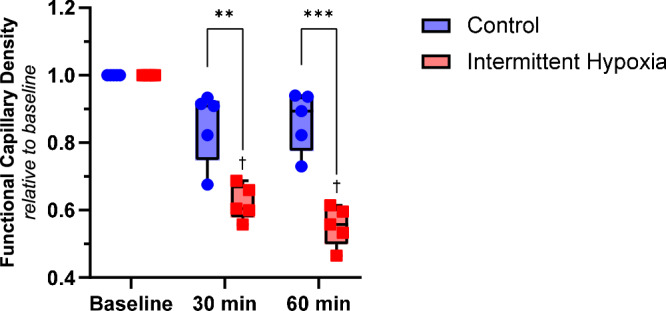
Functional capillary density. Relative changes in functional capillary density (FCD) from baseline measured at baseline, 30 min, and 60 min during exposure to control (21%/21% O₂) or intermittent hypoxia (21%/10% O₂) conditions. Oxygen was cycled every 30 s and delivered directly into the restraining tube at a flow rate of 1 L/min. Functional capillary density is reported relative to baseline to account for intrinsic spatial heterogeneity of capillary perfusion and to emphasize dynamic changes in capillary recruitment. Data are displayed as box-and-whisker plots, where the center line represents the median, the box denotes the interquartile range (25th–75th percentiles), and the whiskers indicate the 5th–95th percentiles. Intermittent hypoxia significantly reduced FCD compared with both baseline and control conditions. Each group consisted of 5 animals, with at least 12 fields analyzed per animal. Statistical analyses were performed using mean values of each animal.† indicates significant difference from baseline, and * (*P* < 0.05), ** (*P* < 0.01) indicate significant differences from control.

The FCD, a critical indicator of tissue perfusion and determined by the perfused capillary length per tissue area, show an statistically significant decrease in the IH group at both 30 and 60 mins compared to baseline and control (Fig. 5). This reduction suggests a reduction in microvascular capillary perfusion pressure, associated with the reduction of vascular blood flow. In contrast, the control group maintained consistent FCD throughout the observation period, indicating preserved microcirculatory perfusion.

## Oxygen saturation dynamics and hyperspectral imaging

**Fig. 6:**
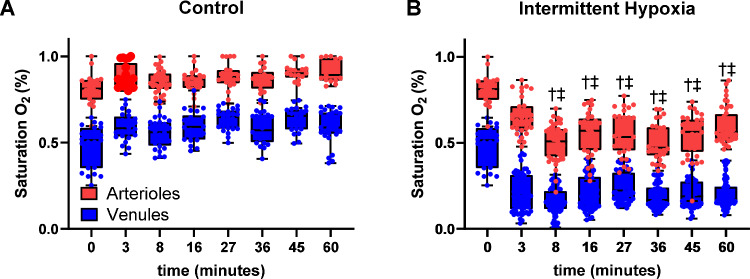
Saturation O_2_—oxygen saturation (O₂ %) dynamics in arterioles and venules under control and intermittent hypoxia conditions over a 60-min observation period. **A** Control group shows stable oxygen saturation levels in both arterioles and venules. **B** Intermittent hypoxia group shows a marked decline in oxygen saturation in both vessel types. Arterioles and venules of the intermittent hypoxia group were fitted with a one-phase decay model, yielding rate constants of 0.398 min^−^¹ for arterioles and 1.014 min^−^¹ for venules. Data are displayed as box-and-whisker plots, where the center line represents the median, the box denotes the interquartile range (25th–75th percentiles), and the whiskers indicate the 5th–95th percentiles. Control group includes 30 vessels per time point, and intermittent hypoxia group includes 45 vessels per time point. Statistical analyses was performed using mean values. †, indicates significant difference from baseline for the arterioles (*P* < 0.05) ‡, indicates significant difference from baseline for the venules (*P* < 0.05).

**Fig. 7:**
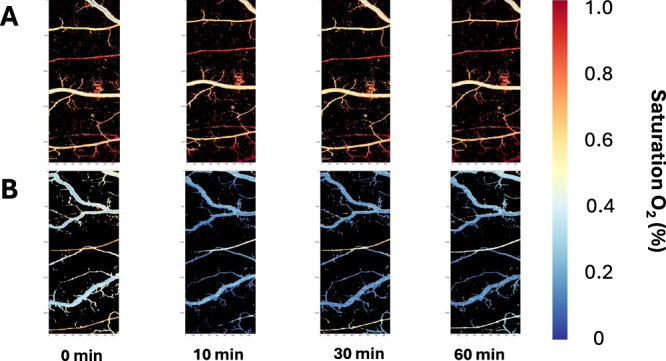
Hyperspectral images of the dorsal window chamber’s microcirculation. **A** Control group (21%/21% O₂) and **B** Intermittent hypoxia group (21%/10% O₂), shown at baseline, 10 min, 30 min, and 60 min. Images were captured every minute for the first 10 min and every 3 min thereafter for a total of 60 min. Color-coded maps represent oxygen saturation (SO₂) values across the vascular network, with corresponding scale on the right. Image analysis methods were adapted from ref. ^22^.

Hyperspectral imaging revealed rapid and sustained declines in microvascular Hb SO₂ in the IH group (Figs. 6 and 7). Arteriolar and venular Hb SO₂ both decreased significantly within the first 10 minutes of exposure to IH, stabilizing at new lower set points after approximately 8 min. Fitting the microvascular SO_2_ to a one-phase exponential decay model yielded a rate constant of 0.4 min^−1^ for arterioles and 1.0 min^−1^ for venules, illustrating the greater sensitivity of venous compartments to hypoxic stress. These changes were absent in the control group, where SO₂ remained stable throughout the 60-min observation period. Representative hyperspectral images of microvascular SO_2_ further demonstrated the spatial distribution of Hb SO_2_s across the microvascular network, highlighting large regions of tissue with diminished oxygenation in the IH group compared to control group.

### Oxygen transport, delivery and extraction

**Fig. 8:**
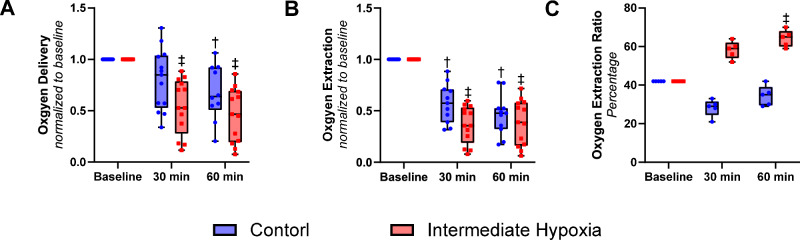
Oxygen transport dynamics during exposure to control (21%/21% O₂) or intermittent hypoxia (21%/10% O₂) in animals instrumented with the dorsal window chamber model. Oxygen was cycled every 30 s and delivered directly into the restraining tube at a flow rate of 1 L/min. **A** Oxygen delivery (control baseline: 0.14 ± 0.08 µL/min; intermittent hypoxia baseline: 0.11 ± 0.05 µL/min), **B** oxygen extraction (control baseline: 0.06 ± 0.03 µL/min; intermittent hypoxia baseline: 0.04 ± 0.02 µL/min), and **C** oxygen extraction ratio (fraction of delivered O₂ extracted) were calculated at baseline (BL), 30 min, and 60 min. Data are displayed as box-and-whisker plots, where the center line represents the median, the box denotes the interquartile range (25th–75th percentiles), and the whiskers indicate the 5th–95th percentiles. Intermittent hypoxia resulted in marked reductions in both O₂ delivery and extraction, accompanied by a compensatory increase in the O₂ extraction ratio relative to control. Statistical analyses were performed using mean values using every independent vessel for VO_2_ and DO_2_ (*n* = 16) and the mean value using per animal (*n* = 5). †, indicates a significant difference from baseline in the control group (*P* < 0.05); and ‡, indicates a significant difference from baseline in the intermittent hypoxia group (*P* < 0.05).

Analysis of oxygen transport dynamics (Fig. 8) revealed that DO₂ and VO₂ were significantly reduced in the IH group at both 30 and 60 min compared to baseline and the control group. These reductions reflect the combined effects of decreased microvascular blood flow and lower microvascular Hb SO_2_. In contrast, the oxygen extraction ratio (OER) increased significantly under IH, indicating an attempted compensatory response wherein tissues extracted a higher proportion of the available oxygen. Despite this increase, the overall decline in VO₂ and DO₂ underscores the failure of the efforts compensate to meet metabolic demands. Although, the control group exhibited significant changes in DO₂ and VO₂ compared to baseline, the stability of O_2_ transport under normoxic cycling conditions the oxygen extraction ratio remains close to baseline and since the OER is a representation of the tissue’s oxygen utilization through time the control illustrates proper oxygenation utilization.

## Discussion

This study supports the hypothesis that, in response to acute cyclic intermittent hypoxia (21%/10% O₂), compensatory microvascular mechanisms are insufficient to maintain adequate DO_2_, leading to impaired tissue oxygenation. Using hyperspectral imaging, we documented a progressive decline in microvascular Hb SO₂ over the 60-min exposure to cyclic IH. While the Hb SO₂ levels in arterioles and venules remained relatively stable in the control group, animals exposed to IH exhibited a rapid and sustained reduction in Hb SO2 and in blood flow in the microcirculation. The Hb SO_2_ reduction in arterioles followed a one-phase exponential decline, while venules showed a more pronounced decay, underscoring their downstream vulnerability to oxygen depletion. In both compartments, Hb SO₂ appeared to reach a new homeostatic set point after approximately 8 min of exposure to this chronic hypoxic burden. This decline in tissue oxygenation was accompanied by complex microvascular hemodynamic responses, including compensatory lack of vasoconstriction in arterioles, vasoconstrictive responses in venules, reduced blood flow across both vessel types, and a significant decrease in FCD. This pattern loosely mirrors pathophysiological features observed in sleep-disordered breathing, particularly obstructive sleep apnea, through the longevity of hypoxic burden impairing vascular autoregulation and contribute to endothelial dysfunction, inflammation, and oxidative stress^2,3,5^.

At the systemic level, the acute hypoxic burden elicited a compensatory cardiovascular response, characterized by a reduction in mean arterial pressure (MAP) and a transient elevation in heart rate (HR). Although previous studies have reported hypertension during IH^23,24^, the differences observed here likely reflect several factors, including the lack of experimental control of ventilation, physiological variability inherent to conscious animals, and species-specific reflex mechanisms in Golden Syrian hamsters, a fossorial mammal with robust baroreflex and ventilatory responses^25^. Consistent with this interpretation, the presence of hypocapnia reflects enhanced ventilation and may influence systemic vascular tone, but changes in arterial pressure likely reflect the combined effects of altered resistance and reduced blood flow in the microvasculature.

In contrast to systemic hemodynamics, the microvasculature exhibited heightened sensitivity due to the acute hypoxic burden, something previously reported^26–28^. As the duration of this hypoxic exposure increased, modulation of carotid body and peripheral chemoreceptor activity likely occurred^23,24^. As the duration of the acute hypoxic burden progressed, activation of more peripheral chemoreceptors, including the carotid bodies, likely contributed to continuing coordinated microvascular adjustments^29,30^. These oxygen-sensing pathways initiate signaling cascades that promote redistribution of blood flow and optimization of tissue oxygen utilization in response to reduced oxygen availability^27,31^. The accompanying changes in plasma electrolytes, such as reduced potassium levels, further support the presence of hypoxia-driven physiological adaptations that may be linked to repeated IH–induced chemoreceptor activation.

Within this context, our findings suggest that even acute IH may be sufficient to perturb vascular function and oxygen transport. Prior studies examining chronic IH have reported alterations in nitric oxide signaling, increased oxidative stress, and elevated inflammatory mediators—processes known to influence microvascular oxygen delivery^26,31,32^. In our study, even in this acute IH setting microvascular diameters increased while blood flow decreased, suggesting a microvascular remodeling. One potential explanation for this pattern is hypoxic vasodilation, a compensatory response whereby blood vessels dilate in response to reduced oxygen availability to preserve tissue oxygenation^15,33^. However, it is also possible that the apparent increase in diameter reflects a relative lack of vasoconstrictive tone during the IH-induced reduction in MAP, rather than active dilation per se. Regardless of the underlying mechanism, the persistence of reduced microvascular flow, reduced capillary perfusion, and increased vascular resistance despite these diameter changes indicates that such compensatory mechanisms may be insufficient to normalize perfusion under IH conditions.

While the present findings are attributed to acute, chemoreceptor-mediated microvascular responses to hypoxic burden, extensive prior work has characterized the longer-term consequences of sustained or recurrent hypoxia on blood rheology and blood flow^33–35^. Severe chronic hypoxic exposure has been shown to affect multiple blood components, including white blood cells, thrombocytes, and non-cellular elements^36^. Chronic IH has been associated with increased plasma viscosity, enhanced erythrocyte and thrombocyte aggregation, and elevated fibrinogen levels^36,37^. Ultimately, these perturbations associated with any type of hypoxia effects the whole blood environment negatively impacting blood flow as a whole, but particularly microvascular flow, a key marker of oxygen offloading^38^. Consistent with this framework, our findings of reduced microvascular flow following acute cyclic IH suggest that repeated engagement of oxygen-sensing and chemoreceptor-mediated compensatory pathways may, if chronically invoked, predispose the microcirculation to lasting structural and functional alteration.

Interestingly, the control group also exhibited signs of vascular regulation, particularly by the 60-minute mark. Despite normoxic conditions, an increase in vascular resistance was observed in peripheral tissues raising the possibility of an autoregulatory or oxygen feedback loop responding to sustained high oxygen availability. Something that has been previously reported as a key function of the microcirculation to prevent oxidative damages to the tissues as a result of increased oxygen concentration^38^. Moreover, the oxygen extraction ratio in the peripheral tissue remained at a comparable level to baseline, prompting speculation that tissues oxygen utilization is being maintained in the control regardless of the phenomena seen in the microvascular bed.

Our findings support prior work suggesting that an increased oxygen extraction ratio does not necessarily arise from inadequate systemic oxygen delivery (DO₂), but rather from heterogeneous microcirculatory blood flow. In this context, microvascular dysfunction can lead to uneven perfusion, whereby some regions receive excess flow while others are under perfused, resulting in impaired effective DO₂ to peripheral tissues^12,39,40^. As a consequence, peripheral tissues may experience persistent microregional hypoxia driven by oscillatory oxygen exposure rather than sustained reductions in systemic DO₂. In the context of chronic obstructive sleep apnea, where such oxygen oscillations occur nightly, this framework provides a physiological basis for understanding how hypoxic burden and oxygen cycling may progressively impair microcirculation and tissue-level oxygen availability.

## Conclusion

In summary, this study establishes a mechanistic foundation for understanding how repeated hypoxic exposures acutely disrupt microvascular oxygen delivery, perfusion, and capillary function. By resolving vessel-level and capillary-level responses in real time, the present work demonstrates that even short-duration intermittent hypoxia produces persistent microvascular oxygen deficits that are somewhat compensated by local vascular adaptations. These observations provide a physiological framework for interpreting how chronic hypoxic burdens, such as those encountered in obstructive sleep apnea, may progressively impair microvascular function through repeated oxygen oscillations. Collectively, this experimental platform enables future studies to directly link cumulative hypoxic exposure to long-term microvascular remodeling, dysfunction, and metabolic vulnerability.

## Limitation

It should be discussed that these observations were made in conscious animals, which allows preservation of intact reflexes and physiological regulation but also introduces the possibility that conscious stress responses may contribute to systemic hemodynamic changes during IH exposure. While obstructive sleep apnea is inherently an unconscious phenomenon, the use of a conscious model may accentuate ventilatory, cardiovascular, or neurohumoral responses that would otherwise be blunted under anesthesia. This represents a potential limitation when interpreting systemic outcomes; however, it also strengthens the relevance of the microvascular findings, which reflect integrated responses occurring in the presence of intact neural and vascular control mechanisms. Additionally, only male hamsters were used in this study, and sex-related differences in vascular regulation, hormonal signaling, and hypoxic responses may influence microvascular and systemic adaptations to intermittent hypoxia. Therefore, the findings presented here may not fully capture potential sex-dependent physiological responses. Future studies incorporating both sexes, as well as controlled ventilation or sleep-state modeling, will be valuable for further dissociating hypoxia-driven effects from conscious stress responses.

## Data sharing availability

Numerical source data can be found in Supplementary Data 1.

## Supplementary information

Description of Additional Supplementary Files

Supplementary Data 1

Reporting Summary

Transparent Peer Review file
