# Supplementary material for: Intermittent Hypoxia Associated with Sleep Apnea Disrupts Microvascular Hemodynamics and Oxygen Delivery
Source: Commun Biol. 2026 Apr 11;9:822. doi: 10.1038/s42003-026-10027-z (PMC13269800; doi:10.1038/s42003-026-10027-z)
Supplement: Supplementary file 1 — Description of Additional Supplementary Files [file 42003_2026_10027_MOESM1_ESM.docx]

**Description of Additional Supplementary Files**

**File name:** Supplementary Data 1

**Description:** Numerical source data for all graphs in the manuscript.
