# Supplementary material for: Intermittent Hypoxia Associated with Sleep Apnea Disrupts Microvascular Hemodynamics and Oxygen Delivery
Source: Commun Biol. 2026 Apr 11;9:822. doi: 10.1038/s42003-026-10027-z (PMC13269800; doi:10.1038/s42003-026-10027-z)
Supplement: Supplementary file 4 — Transparent Peer Review file [file 42003_2026_10027_MOESM4_ESM.pdf]

nature portfolio

Peer Review File

Intermittent Hypoxia Associated with Sleep Apnea Disrupts Microvascular Hemodynamics and Oxygen Delivery

Corresponding Author: Professor Pedro Cabrales

This file contains all reviewer reports in order by version, followed by all author rebuttals in order by version.

Version 0:

Reviewer comments:

Reviewer #1

(Remarks to the Author)
In this manuscript, the authors use a hamster model instrumented with an implanted window to allow intravital microscopy on the conscious restrained animal. The hamsters were exposed to alternate periods (30s) of hypoxic gas (10% O2) over a 1-hour period whilst the tissue below the window was visualised. In response to intermittent hypoxia (IH), there was a significant sustained fall in the observed arterial PO2 and consequently in the Hb saturation. Data is also presented showing changes in the microcirculation and oxygen transport dynamics during IH. These were compared to observations made in control animals using the same protocol except for alternating between normoxic air supplies each 30s.

The authors have a track record of using this animal preparation and for using HSI to monitor the microcirculation. The experiments were carried out following local ethical review and approval. The ability to visualise the microcirculation without anaesthesia is of interest to the wider community, as is understandings OSA effects due to increasing prevalence.

The major findings in this manuscript are that, despite the hypoxic periods being intermittent, there is a sustained fall in arterial PO2 to a new steady state level resulting in a sustained hypoxic stimulus. Additionally, the authors demonstrate the use of HSI for monitoring microvascular saturation and the sustained effects of IH. The authors suggest that these approaches are useful to study O2 kinetics in this model of OSA and may be helpful in targeting future approaches at maintaining microcirculatory function.

Comments

Methods

1. Were the hamsters acclimatised to the restraining tool before the measurement protocols were used? It states a 20min to adjust, however, other approaches using restraint on conscious animals (eg tail cuff blood pressure measurements) may acclimatise over several days before starting the protocol to reduce any acute stress responses by being restrained.

2. Have the authors assessed the impact of a carotid artery cannulation on the function of the carotid body from ipsilateral side of the hamster? In OSA, carotid bodies are often considered to be important in driving systemic pathology such as hypertension.

3. Inclusion criteria for mice is a PO2 of 70mmHg whereas for hamsters it is 50mmHg. Since hamsters are used then it would be better to use an inclusion criteria for hamsters – eg. Torres Filho (1996) where this group demonstrated that PO2 in the hamster skinfold preparation is lower than systemic PO2.

4. The Statistical Analysis section states results are presented as mean ± SD (and also in some figure legends), however, most are plotted as box and whisker – with no description of what the box and whisker components are plotted as. The authors should explain the layout (eg is the mean or median plotted? What are the box edges and whiskers denoting?)

Results

1. Where results are plotted relative to a baseline value, the inclusion of the absolute values (mean ± SD) for the baselines would help the reader better understand the physiological changes from baseline.

2. The text states that lactate levels increased significantly in the IH group after 60 min (Table 2), however, Table 2 shows the peak value at 30 min and no indication of a statistically significant difference at either 30 or 60 min in the Table. Can the authors comment/clarify this please? Elevated lactate levels are mentioned in the discussion also.

3. Figure 1 - the standard way to write blood gases would be PaO2 (as in Table 1) and not pO2,A – the same applies to other blood gas parameters. Figure 1 legend – the microcirculatory metric measured would be red blood cell velocity not vessel velocity?

4. Figure 2 shows separate BL values for the Control and IH group, however, Table 1 has them combined. For consistency, it would be best to have them separate in Table 1 also.

5. Figure 3 – this legend states that IH significantly reduced arteriolar diameter and flow compared to baseline and control – whilst Panel A clearly shows IH inducing an increased in diameter above baseline and control. The text states dilation – but also says that it is more pronounced in larger arterioles (Panel B) whereas it appears more pronounced in Panel A?

6. Figure 4 – this figure is confusing when considered with the description of the resistance calculation given in the Methods (R=P/Q). In the IH group, the MAP falls to about 80% of baseline (Table 1) and the flow falls to about 50% of baseline (Figure 3E) – this would not equate to a fall in resistance to about 50% of baseline. Could the authors please explain this difference.

7. Figure 8 – panel C appears to be missing the error bars from the calculation of OER in the different groups.

Discussion

1. The authors suggest that, at the systemic level, the fall in MAP and increase in HR indicates an autonomic dysregulation. Have they considered the role of hyperventilation (Table 1 shows hypocapnia is induced) and/or the baroreflex in mediating this rather than it being a dysregulation? At what PaO2 are hamster carotid bodies stimulated?

Conclusion

1. The authors conclude that there is compensatory arteriolar dilation with venular constriction – Figure 4C shows venule resistance also decreased during IH but to a lesser extent than control at 60min. This is more of a reduced dilation than a constriction?

2. I am not sure how the authors extrapolate the nightly IH cycles in OSA to ischemia-reperfusion injury per se. There is definitely hypoxia and reoxygenation and OSA patients may be more at risk of stroke or MI but whether OSA itself would be inducing ischemia-reperfusion injury is debatable.

Minor comments

1. There are typos throughout the MS such as incorrect opening/closing of brackets or word order (eg the first sentence starts “Sleep breathing disordered” whereas the accepted condition is “Sleep disordered breathing”). These can easily be rectified by careful proof reading.

2. Many of the figures are labelled with “Intermediate Hypoxia” rather than “Intermittent Hypoxia”

Overall, this MS presents some interesting results on the behaviour of the microcirculation. It is interesting that the IH protocol induced a sustained level of hypoxia in this model – this would increase the hypoxic burden (HB) overall and a recent systematic review concluded that HB was a better CV risk parameter than traditional parameters such as AHI (Coso et al, 2024). The authors may wish to link into this aspect as they propose microvascular dysregulation in OSA.

Reviewer #2

(Remarks to the Author)
I commend the authors on their detailed and thoughtful work in detecting a potentially harmful ischemia reperfusion cycle that evades systemic compensatory mechanisms in a model of obstructive sleep apnea. Suggested revisions are mostly minor.

Introduction
1. Line 1 “sleep breathing disordered” did you mean Sleep breathing disorders? Or Disordered sleep breathing?
2. Line 3, adjust text color to black.
3. Line 7 try “triggers physiological changes in …”
4. End of introduction: You could include systemic compensatory adjustments in your statement of “compensatory microvascular adjustments are insufficient” since you report cardiovascular parameters and blood chemistry.
Methods
1. Instead of “approved by local animal care committee” state the IACUC approval number.
Results
1. Changes in Microvascular Resistance: Contains a lot of discussion material. A result’s purist would suggest sticking to the facts about microvascular resistance and discussing the “paradox” later. Also:
You state that “despite the reductions in MAP and blood flow, vascular resistance decreased.” An alternate explanation that fits with your microvascular narrative is that arteriolar dilation, and decreased vascular resistance, are responsible for the decrease in MAP.
2. Calculation for OER is not described in methods or results.
Discussion:
1. “at the systemic level … significant reduction in MAPT and initial elevation of HR, indicative of autonomic dysregulation.” Dysregulation indicates the cardiovascular system is responding abnormally. The system appears to be responding normally to a mild and sustainable shift to anerobic respiration (as evidenced by IH lactic doubling from BL and holding steady throughout the IH protocol) brought on by decreased DO2.
Suggest toning down the paragraph from “overwhelmed” to focus on the observation that the IH CV system is sufficiently stressed to raise risk for the downstream effects you describe in the next paragraph. The language used in the conclusion conveys the appropriate perspective.
2. The use of conscious animals is commendable and often missing in oxygen transport studies. However, where we often want to study conscious events (exercise, injury response, etc) and use anesthetized models, here you are studying an unconscious phenomenon using a conscious model. Suggest discussing whether this is a limitation to the study, and if there is a conscious stress component that may affect systemic responses to the IH protocol.

Reviewer #3

(Remarks to the Author)
COMMSBIO-25-9534
This manuscript details the results from a study investigating the systemic hemodynamic and local microvascular responses to acute intermittent hypoxia in hamsters. The authors argue that this model has relevance to sleep disordered breathing. Overall, the study appears to have been well performed and the results and discussion reflect the underlying data. Making these measures in the un-anaesthetized state is tricky and the authors should be commended for their overall study design. Despite these positive points, I do have some suggestion for improving clarity as well as a suggestion for an additional experimental group.

Specific comments
A major theme throughout this manuscript is that acute intermittent hypoxia (AIH) causes vasodilation in the microvasculature as a potential compensatory response to help drive increases in blood flow to maintain oxygen delivery. It is argued that under hypoxic conditions (see constraints on this in comments below) these compensatory mechanisms ultimately fail and despite attempts to extract more oxygen hypoxia is essentially associated with microvascular dysfunction, that may have relevance to what is observed clinically with OSA. Yet, in OSA (or in human lab studies where AIH or AIHH is delivered) there is NOT a corresponding drop in blood pressure. Conversely, in this manuscript (and in other rodent papers too), hypoxia causes a significant drop in MAP. Hence, it is not clear to me if the downstream local microvascular dysfunction reported here is simply caused by a reduction in MAP associated with the hypoxia, or if the impaired responses are due to the hypoxia itself. This could be experimentally tested in one of two ways. Either you could infuse phenylephrine during the 30s hypoxic cycles to hold MAP at baseline – as others have done in rodent studies. Or you could experimentally mimic the repetitive drops in MAP without the hypoxia (for example through repeated hemorrhage whilst observing the drop in BP in real-time). Without one of these two experimental manipulations the reader is left to wonder whether the presented microvascular dysfunction is really due to the hypoxia or is simply a result of reduced driving pressure to reduced MAP.

Minor comments / suggestions
1. Do you know the volume of the restraining tube, and have you checked our quickly the air change engulfs the full tube? This may have implications for how long the animal is actually exposed to the hypoxia for
2. Your MAP data is very high – presumably this is a stress response to the tube. Was there any familiarization of the animals with the tube ahead of the experiment? Telemetric studies from other rodents would suggest MAP should be in the range of 90mmHg.
3. Is the high PaCO2 normal for a hamster?
4. For the vascular resistance measures, how did you estimate local pressure from MAP?
5. You are missing a lot of details from the methods. Please add more details about the animals to your methods. For example, how old were they and how many were there? Also, please rationalize your choice of only male hamsters.
6. Why is your PaO2 changing by ~15% in your control group at 30min.
7. More details about the timepoints of the measurements are needed. If I understand correctly, you are continuously cycling gases in the hypoxia group – if this is the case then when in the cycling do you perform your measurements and over what period of time are they made? I assume they are made during a hypoxia cycle, but if so – how long into a hypoxia cycle as

the whole cycle lasts only 30s? This may explain the quite high variability in your blood gases and MAP data.
8. In many places you write intermediate hypoxia but this should be intermittent hypoxia.
9. Table 1 shows no significant differences in HR but Figure 2 shows increases in HR – which is correct?
10. Please thoroughly check your spelling and grammar throughout as there are a number of small mistakes
11. Since you do not have any direct “read-out” of autonomic functon(i.e., nerve recordings), I would refrain from making inferences about sympathetic outflow, or autonomic failure, in the paper – as this appears to be largely based on your HR data which could be a response to either sympathetic activation, parasympathetic withdrawal, or simply to maintain cardiac output in the face of reducing stroke volume.

Version 1:

Reviewer comments:

Reviewer #1

(Remarks to the Author)
In this revised manuscript the authors have worked through the original comments and provided answers to all the questions raised as well as amending the manuscript where appropriate.

These amendments have improved the clarity of the manuscript and so significantly improved the accuracy and quality, however, there still appears to be one area that seems contradictory.

In the Results>Microvascular Hemodynamics section, the description is of significant vasodilation in both small and large arterioles (Figure 3), however, the following section Results>Changes in Microvascular Resistance reports increases in vascular resistance in both small and large arterioles (Figure 4).

The authors may consider that in Figure 3, the IH group are not actually showing arteriolar vasodilation but just showing a lack of constriction (ie the fall in flow is purely dependent on the MAP falling with no diameter change from baseline)? This may solve the apparent contradiction between these results section.

The overall improvements in the manuscript are welcome and understanding the behaviour of the microcirculation under different conditions is important to our understanding of the cardiovascular consequences of conditions such as OSA.

Reviewer #2

(Remarks to the Author)
Great work! Concerns addressed.

Reviewer #3

(Remarks to the Author)
Thank you for your responses to my suggestions.

I have only one comment left and that is that the limitations should also specifically identify that only male hamsters were used and that responses may differ in female hamsters.

Open Access This Peer Review File is licensed under a Creative Commons Attribution 4.0 International License, which permits use, sharing, adaptation, distribution and reproduction in any medium or format, as long as you give appropriate credit to the original author(s) and the source, provide a link to the Creative Commons license, and indicate if changes were made.
In cases where reviewers are anonymous, credit should be given to 'Anonymous Referee' and the source.
The images or other third party material in this Peer Review File are included in the article's Creative Commons license, unless indicated otherwise in a credit line to the material. If material is not included in the article's Creative Commons license and your intended use is not permitted by statutory regulation or exceeds the permitted use, you will need to obtain permission directly from the copyright holder.
To view a copy of this license, visit https://creativecommons.org/licenses/by/4.0/

Manuscript Number: COMMSBIO-25-9534.R1

Title: Hemodynamics and Microvascular Oxygenation Under Intermittent Hypoxia to Simulate Sleep Apnea

Dear Editor and Reviewers,

We sincerely thank the Editor and the Reviewers for their careful evaluation of our manuscript and for the detailed, constructive feedback. We appreciate the time and effort invested by you and the reviewers. We are grateful for the constructive and thorough feedback provided.

We have carefully considered all reviewer comments, with particular attention to clarifying the methodological approach, addressing animal acclimatization, and effects of hypoxia. We appreciate the recognition of studying a complex topic in an unanesthetized animal. We thank you for all your comments and recommendations, we all are very grateful as many aspects of the manuscript have improved in clarity, methodological transparency, and interpretation after implementing your recommendations and suggestions.

In response, we have undertaken substantial revisions to address all comments raised.

Below, we respond point by point to the Editor's requirements and to each Reviewer's comments. All changes have been incorporated into the revised manuscript and are clearly marked in red, and responses are in *blue italics*. Revised version includes several clarifications added to the Introduction, Methods, Results, Discussion, and a newly strengthened Limitations section.

Thank you,

All authors,

Reviewers' comments:

Reviewer #1 (Remarks to the Author):

In this manuscript, the authors use a hamster model instrumented with an implanted window to allow intravital microscopy on the conscious restrained animal. The hamsters were exposed to alternate periods (30s) of hypoxic gas (10% O2) over a 1-hour period whilst the tissue below the window was visualized. In response to intermittent hypoxia (IH), there was a significant sustained fall in the observed arterial PO2 and consequently in the Hb saturation. Data is also presented showing changes in the microcirculation and oxygen transport dynamics during IH. These were compared to observations made in control animals using the same protocol except for alternating between normoxic air supplies each 30s.

The authors have a track record of using this animal preparation and for using HSI to monitor the microcirculation. The experiments were carried out following local ethical review and approval. The ability to visualize the microcirculation without anesthesia is of interest to the wider community, as is understandings OSA effects due to increasing prevalence.

The major findings in this manuscript are that, despite the hypoxic periods being intermittent, there is a sustained fall in arterial PO2 to a new steady state level resulting in a sustained hypoxic stimulus. Additionally, the authors demonstrate the use of HSI for monitoring microvascular saturation and the sustained effects of IH. The authors suggest that these approaches are useful to study O2 kinetics in this model of OSA and may be helpful in targeting future approaches at maintaining microcirculatory function.

Thank you very much for the time and effort you devoted to reviewing our manuscript. We sincerely appreciate your careful evaluation and the thoughtful suggestions and recommendations you provided. Your comments were constructive and insightful, and they helped us identify key areas where the manuscript needed strengthening. We have carefully considered each of your points and revised the manuscript accordingly to improve the clarity of the methodology and the overall quality of the work.

Comments

Methods

1. Were the hamsters acclimatised to the restraining tool before the measurement protocols were used? It states a 20min to adjust, however, other approaches using restraint on conscious animals (eg tail cuff blood pressure measurements) may acclimatize over several days before starting the protocol to reduce any acute stress responses by being restrained.

We thank the reviewer for raising this important point regarding restraint acclimatization and potential stress responses. We agree that insufficient habituation to restraint can confound physiological measurements, particularly in conscious animals.

In the present study, Golden Syrian hamsters underwent a multi-day acclimatization to the restraining tube, rather than being introduced acutely on the day of experimentation. Upon arrival from Charles River Laboratories, animals were housed in cages containing the same restraining tube used during experiments, allowing them to freely explore and habituate the device for a minimum of three days prior to any surgical procedures to implant the dorsal window and the catheters. Importantly, Golden Syrian hamsters are a fossorial species with a natural tendency to seek enclosed, tunnel-like (burrow) environments for shelter, nesting, safety and burrowing. As such, the restraining tube used to house the animal during observation represents a familiar space and ethologically relevant structure, which facilitates voluntary entry and limits the stress during the intermittent hypoxic challenge.

Following the tube acclimatization period, animals underwent dorsal window chamber implantation and subsequent catheterization. At this stage, the restraining tube was removed from the home cage to prevent accidental interference or injury to the window chamber or vascular catheters. Experiments were conducted one day after catheterization, corresponding to approximately two days without interaction with the restraining tube.

The 20-minute period described in the Methods refers specifically to a brief stabilization period after placement into the restraining tube on the microscope stage and does not represent the primary acclimatization phase. This interval allowed animals to settle following transfer to the experimental setup and ensured stable baseline systemic and microvascular parameters prior to the data acquisition.

This combined approach, home-cage habituation over several days to an ethologically relevant enclosure, followed by a short pre-measurement stabilization period, was designed to minimize acute restraint-induced stress and is consistent with established protocols for other conscious animal models. We have revised the Methods section to explicitly clarify the acclimatization strategy.

To address the reviewer's comment, we have revised the methods of the manuscript including the following passage in the animal preparation section:

To minimize restraint-related stress during conscious measurements, the animals were acclimatized to the restraining tube prior to experimentation. Upon arrival from animal provider, the same tube used during imaging was placed in the home cage for at least three days to allow voluntary exploration and habituation; this approach leverages the species' natural preference for enclosed, burrow-like environments. The tube was removed after window chamber implantation and catheterization to prevent interference with the surgical preparation. Experiments were conducted one day after catheterization to ensure catheter patency.

2. Have the authors assessed the impact of a carotid artery cannulation on the function of the carotid body from ipsilateral side of the hamster? In OSA, carotid bodies are often considered to be important in driving systemic pathology such as hypertension.

We thank the reviewer for this important question. Carotid body function was not directly assessed following carotid artery cannulation in this study. However, several features of the experimental design minimize the likelihood that ipsilateral carotid body impairment confounded the results.

Internal carotid catheterization was performed using established microsurgical techniques that preserve arterial patency, and animals were allowed to recover prior to experimentation. Only animals with stable baseline systemic parameters (MAP, HR, and blood gases) were included, indicating preserved autonomic and chemoreflex function at baseline. Moreover, hypoxic cardiovascular responses are mediated by integrated signaling from central chemoreceptors, the contralateral carotid body, and aortic bodies;[1,2] therefore, systemic hypoxic responses are not solely dependent on a single carotid body.

Importantly, both experimental and control groups underwent identical surgical preparation, such that any potential alteration in carotid body signaling would be present in both groups and would not bias group comparisons. As the primary focus of this study was to document the acute microvascular hemodynamics and oxygen transport during intermittent hypoxia, rather than chronic chemoreflex-driven pathology. We agree with the reviewer, that future studies will explored the microvascular and oxygen changes created by chronic intermittent hypoxia.

(1) Kumar, P. Sensing Hypoxia in the Carotid Body: From Stimulus to Response. *Essays in Biochemistry* **2007**, *43*, 43–60. https://doi.org/10.1042/bse0430043.
(2) Guyenet, P. G. Regulation of Breathing and Autonomic Outflows by Chemoreceptors. *Comprehensive Physiology* **2014**, *4* (4), 1511–1562. https://doi.org/10.1002/j.2040-4603.2014.tb00589.x.

3. Inclusion criteria for mice is a PO2 of 70mmHg whereas for hamsters it is 50mmHg. Since hamsters are

used then it would be better to use an inclusion criteria for hamsters – eg. Torres Filho (1996) where this group demonstrated that PO2 in the hamster skinfold preparation is lower than systemic PO2.

We thank the reviewer for this important clarification and agree that inclusion criteria should be species-specific and grounded in established physiology for the hamster physiology and the dorsal window preparation.

As demonstrated by Torres Filho et al. (1996), oxygen tension within the hamster dorsal skinfold microcirculation is physiologically lower than systemic arterial pO_2 due to longitudinal oxygen loss along the arteriolar network. Additionally, hamster dorsal skinfold microcirculation shows similar pO2 distributions to other rodents (mice and rats). First-order arterioles exhibit substantially reduced pO_2 relative to systemic arterial values, with further decreases toward terminal arterioles, capillaries, and venules. These microvascular gradients are a well-recognized and normal feature of the cardiovascular system.

We note, however, that arteriolar pO_2 reflects local microvascular oxygen distribution and it is very specific to the arteriolar size and branching, thus, it might not be the best parameter to define the inclusion criterion for animal health. Accordingly, we have used systemic arterial pO_2 during baseline as screening parameter, consistent with standard practice in conscious animal studies. An arterial pO_2 inclusion threshold of ≥ 50 mmHg at baseline is consistent with reported physiological ranges in awake Golden Syrian hamsters.

We have revised the Methods section to explicitly state that systemic arterial pO_2 inclusion criteria were selected based on hamster-specific physiology and the known reduction from systemic to microvascular pO_2 within the dorsal skinfold preparation, as described by Torres Filho et al. (1996).

We've included the following sentence to strength the manuscript

Inclusion criteria for arterial pO_2 (≥ 50 mmHg) were selected to reflect hamster-specific respiratory physiology. Hamsters exhibit fossorial (burrowing) behaviors and unique physiological adaptations that allow them to tolerate the low-oxygen (hypoxic) and high-carbon dioxide (hypercapnic) environments found within burrows. However, microvascular pO_2 distribution in the dorsal window chamber model is comparable to the pO2 distribution observed in other rodents including mice and rats instrumented with dorsal windows. [3,4]

(3) Cabrales, P.; Tsai, A. G.; Frangos, J. A.; Intaglietta, M. Role of Endothelial Nitric Oxide in Microvascular Oxygen Delivery and Consumption. *Free Radic Biol Med* **2005**, *39* (9), 1229–1237. https://doi.org/10.1016/j.freeradbiomed.2005.06.019.

(4) Filho, I. P. T.; Kerger, H.; Intaglietta, M. pO2Measurements in Arteriolar Networks. *Microvascular Research* **1996**, *51* (2), 202–212. https://doi.org/10.1006/mvre.1996.0021.

4. The Statistical Analysis section states results are presented as mean ± SD (and also in some figure legends), however, most are plotted as box and whisker – with no description of what the box and whisker components are plotted as. The authors should explain the layout (eg is the mean or median plotted? What are the box edges and whiskers denoting?)

We thank the reviewer for highlighting this point and agree that the presentation of box-and-whisker plots should be explicitly defined for clarity. Normality tests were used to determine if a data set is well-modeled by a normal distribution. In the revised manuscript, we have clarified that box-and-whisker plots were used to illustrate the distribution of nonparametric data.

Specifically, the center line represents the median, the box denotes the interquartile range (25th–75th percentiles), and the whiskers extend from the 5th to the 95th percentiles. Parametric data is presented as line plots and tables, and it is presented as mean ± standard deviation to emphasize temporal trends and summary statistics. Although, medians are shown in box-and-whisker plots for visual clarity. All statistical comparisons were performed using appropriate statistical tests consistent with the distribution of the data (parametric and non-parametric) and using the appropriate statistical methods.

We have revised the Statistical Analysis section and relevant figure legends to explicitly describe the box-and-whisker plot layout, and all statistical methods to ensure statistical rigor, and consistency across figures.

We adjusted the main manuscript to include the following description:

Normality tests were used to determine if a data set is well-modeled by a normal distribution. Results are presented as box-and-whisker plots to illustrate non-parametric data visualization. Box-and-whisker does not assume an underlying statistical distribution and display the median (center line), interquartile range (box; 25th–75th percentiles), and 5th–95th percentiles (whiskers). Parametric results are depicted as mean ± standard deviation, in addition, tabulated data is presented as mean ± standard deviation. Normality was evaluated using Shapiro-Wilk test.

Results

1. Where results are plotted relative to a baseline value, the inclusion of the absolute values (mean ± SD) for the baselines would help the reader better understand the physiological changes from baseline.

We thank the reviewer for this constructive suggestion and agree that reporting absolute baseline values improves physiological interpretation when results are presented relative to baseline. Accordingly, in the revised manuscript we have added absolute baseline values (mean ± SD) to the figure captions for all baseline-normalized variables where absolute units are physiologically meaningful and comparable across animals (e.g., vessel diameter, blood flow, and oxygen transport parameters). For functional capillary density (FCD), baseline values are not reported in absolute units. FCD is inherently dependent on the specific microscopic field analyzed and exhibits substantial spatial heterogeneity even under baseline conditions. Similarly, Interpretation of vascular resistance among different animal species varies significantly due to differences in size, metabolic rates, cardiovascular structure, and regulatory mechanisms, making relative to baseline-normalized resistance a more informative metric.

2. The text states that lactate levels increased significantly in the IH group after 60 min (Table 2), however, Table 2 shows the peak value at 30 min and no indication of a statistically significant difference at either 30 or 60 min in the Table. Can the authors comment/clarify this please? Elevated lactate levels are mentioned in the discussion also.

We sincerely apologize and thank the reviewer for pointing out this inconsistency/error. Upon review, we agree that the original text miswrote the timing and statistical significance of the lactate response in the intermittent hypoxia (IH) group. As correctly noted by the reviewer, Table 2 shows that lactate levels peak at 30 minutes, with not statistically significant noted at 60 minutes. We have revised the Results sections to accurately reflect these findings.

The text in the result section now states that lactate levels increased in the IH group at 30 minutes, followed by a downward trend at 60 minutes, without implying statistical significance at later time points. This revised text aligns the narrative with the data presented in Table 2. The section in the results now reads:

Lactate levels increased in the IH group after 30 mins (Table 2), indicating a shift toward anaerobic metabolism due to insufficient tissue oxygenation. However, lactate levels decrease in the IH group at the 60 mins timepoint.

3. Figure 1 - the standard way to write blood gases would be PaO2 (as in Table 1) and not pO2,A – the same applies to other blood gas parameters. Figure 1 legend – the microcirculatory metric measured would be red blood cell velocity not vessel velocity?

We thank the reviewer for these helpful suggestions and agree with both points.

First, we have revised the notation of blood gas parameters in Figure 1 to follow standard physiological conventions. Specifically, arterial and venous oxygen partial pressures are now reported as PaO_2 and PvO_2,

respectively, and the same standardized notation has been applied consistently to all other blood gas parameters to match the terminology used in Table 1.

Second, we agree that the appropriate microcirculatory metric measured in this study is red blood cell (RBC) velocity rather than vessel velocity. We have corrected the Figure 1 legend (and schematic labels where applicable) to explicitly refer to RBC velocity, ensuring accurate description of the measured parameter.

4. Figure 2 shows separate BL values for the Control and IH group, however, Table 1 has them combined. For consistency, it would be best to have them separate in Table 1 also.

We thank the reviewer for noting this inconsistency. For clarity and consistency with Figure 2, we have revised Table 1 to report baseline (BL) values separately for the Control and intermittent hypoxia (IH) groups rather than combining them. This change allows direct comparison across figures and tables and improves transparency of group-specific baseline values.

5. Figure 3 – this legend states that IH significantly reduced arteriolar diameter and flow compared to baseline and control – whilst Panel A clearly shows IH inducing an increased in diameter above baseline and control. The text states dilation – but also says that it is more pronounced in larger arterioles (Panel B) whereas it appears more pronounced in Panel A?

We apologize and thank the reviewer for carefully identifying this inconsistency. We agree that the original figure legend and text were inaccurate and did not correctly reflect the data shown in Figure 3.

As correctly noted by the reviewer, intermittent hypoxia (IH) induces arteriolar dilation rather than constriction, with diameters exceeding baseline and control values. We have therefore revised the Figure 3 legend to remove the statement that IH reduced arteriolar diameter and to explicitly state that IH increased arteriolar diameter relative to both baseline and control.

With respect to vessel size, we also agree that the magnitude of the dilation is more pronounced in small arterioles (<60 μm; Panel A) compared with large arterioles (>60 μm; Panel B), as evidenced by the greater relative increase above baseline in Panel A. However, the results meant to reflect the level of significance compared to the control was more pronounced in the larger arterioles than the smaller arterioles. The result description has been revised to clarify the observation in smaller and larger arteriolar vascular tone changes in response to intermittent hypoxia.

We have striven to resolve the discrepancy between the figures, legends, and Results text, to accurately describe the size-dependent arteriolar responses during intermittent hypoxia.

Revised manuscript:

Small arterioles (<60 μm in diameter) demonstrated a significant vasodilation after 60 minutes in the IH group compared with the control group. Similarly, large arterioles (>60 μm) also exhibited significant vasodilation under IH conditions at 60 minutes; however, the magnitude of the difference relative to control was more pronounced in large arterioles than in small arterioles.

6. Figure 4 – this figure is confusing when considered with the description of the resistance calculation given in the Methods (R=P/Q). In the IH group, the MAP falls to about 80% of baseline (Table 1) and the flow falls to about 50% of baseline (Figure 3E) – this would not equate to a fall in resistance to about 50% of baseline. Could the authors please explain this difference.

We thank the reviewer for pointing out this inconsistency in the results and apologize for the confusion. The Methods section correctly defines microvascular resistance as $R = \Delta P/Q$ (here approximated as $R = MAP/Q$). Upon checking our calculations for the resistance panel, we found that the values plotted were inadvertently computed as Q/MAP (i.e., microvascular conductance, the inverse of resistance) rather than MAP/Q. This

*inversion explains why the plotted response appeared inconsistent with the expected relationship: in the intermittent hypoxia group, MAP decreases to ~80% of baseline while flow decreases to ~50% of baseline, which would be expected to **increase** resistance (because $R \propto \mathrm{MAP}/Q$), not to decrease.*

We have corrected the calculation and updated the figure (and corresponding result text and figure legend). Current resistance metric is consistently reported as MAP/Q, normalized to baseline. This correction resolves the apparent inconsistency and aligns the plotted data with the Methods description and the physiological interpretation.

7. Figure 8 – panel C appears to be missing the error bars from the calculation of OER in the different groups.

We thank the reviewer for pointing this error. The error bars in Figure 8C are now clearly visible in the revised version.

Discussion

1. The authors suggest that, at the systemic level, the fall in MAP and increase in HR indicates an autonomic dysregulation. Have they considered the role of hyperventilation (Table 1 shows hypocapnia is induced) and/or the baroreflex in mediating this rather than it being a dysregulation? At what PaO2 are hamster carotid bodies stimulated?

We thank the reviewer for this insightful comment and agree that the observed systemic responses should not be interpreted as autonomic dysregulation. As noted, hypocapnia was observed during intermittent hypoxia (Table 1) and it is indicative of changes in ventilation, which together with baroreflex-mediated cardiovascular adjustments, likely contributes to the observed reduction in mean arterial pressure (MAP) and transient elevation in heart rate (HR). Importantly, animals were free breeding, and ventilation was not experimentally controlled in this conscious animal model, and therefore hyperventilation represents a plausible and expected physiological contributor to the systemic hemodynamic responses.

In response to this comment, we have revised the manuscript to shift the interpretation from autonomic dysregulation to integrated sensory and reflex-driven responses. The revised text now emphasizes the combined roles of ventilatory changes, baroreflex mechanisms, and peripheral chemoreceptor activation in shaping cardiovascular responses during acute intermittent hypoxic. The reduction in PaCO□ is consistent with hyperventilation-induced vasodilation and reduced systemic vascular resistance, while baroreflex engagement in conscious animals may further affect the HR and MAP.

With respect to oxygen sensing, carotid body chemoreceptors in hamsters are expected to be activated at arterial PaO□ values below ~60 mmHg (https://doi.org/10.1152/jappl.2000.88.6.228), with increasing activation as PaO□ declines further. Given the severe arterial hypoxemia achieved during repeated intermittent hypoxia cycling, peripheral chemoreceptor activation is likely and may contribute to both systemic and microvascular adaptations observed in this study.

We have revised the Discussion accordingly to clarify that the systemic cardiovascular changes observed during acute intermittent hypoxia reflect adaptive physiological responses to hypoxic stress, rather than primary autonomic dysfunction. The discussion of the manuscript now includes the following adjusted passage:

In contrast to systemic hemodynamics, the microvasculature exhibited heightened sensitivity to acute hypoxic stress, particularly within hypoxic tissue environments.[25,29,30] The duration of this acute hypoxic challenge begins to trigger carotid body and chemoreceptor modulations.[26,27] As the duration of the acute hypoxic challenge progressed, activation of peripheral chemoreceptors, including the carotid bodies, likely contributed to coordinated cardiovascular and metabolic adjustments.[31,32] These oxygen-sensing pathways initiate signaling cascades that promote redistribution of blood flow and optimization of tissue oxygen in response to reduced oxygen availability.[24,29] The accompanying changes in plasma electrolytes, such as reduced

potassium levels, further support the presence of hypoxia-driven physiological adaptations that may be linked to repeated intermittent hypoxia–induced chemoreceptor activation.

Conclusion

1. The authors conclude that there is compensatory arteriolar dilation with venular constriction – Figure 4C shows venule resistance also decreased during IH but to a lesser extent than control at 60min. This is more of a reduced dilation than a constriction?

We thank the reviewer for this careful observation and apologize for the confusion caused by the earlier version of the figure. Upon re-examination of our analysis, we identified an error in the original vascular resistance calculation (mentioned before), which has now been corrected. Figure 4 has been updated accordingly to reflect resistance calculated as MAP/Q, normalized to baseline.

In the revised figure, intermittent hypoxia (IH) is associated with a significant increase in arteriolar resistance in both small (<60 µm; Panel A) and large (>60 µm; Panel B) arterioles, consistent with reduced effective perfusion despite compensatory changes in vessel diameter. In contrast, venular resistance (Panel C) increased in both groups, with IH producing a greater elevation compared to control. These revised results support the interpretation that IH induces increased downstream microvascular resistance, rather than venular dilation.

2. I am not sure how the authors extrapolate the nightly IH cycles in OSA to ischemia-reperfusion injury per se. There is definitely hypoxia and reoxygenation and OSA patients may be more at risk of stroke or MI but whether OSA itself would be inducing ischemia-reperfusion injury is debatable.

We thank the reviewer for raising this important point and agree that the original wording describing the observation overstated the relationship between intermittent hypoxia and ischemia–reperfusion injury. The present study does not directly demonstrate ischemia–reperfusion injury, nor was it designed to do so.

Accordingly, we have removed references to ischemia–reperfusion injury from the manuscript and revised the Discussion to focus instead on hypoxic burden, intermittent hypoxia via oxygen cycling, and microvascular heterogeneity as mechanistic consequences of intermittent hypoxia. The revised text now frames the clinical relevance more conservatively, emphasizing that intermittent hypoxia–reoxygenation cycles may impose cumulative microvascular perfusion and impair oxygen delivery to tissues, rather than directly inducing ischemia–reperfusion injury.

These revisions align the interpretation with the data presented and avoid extrapolation beyond the scope of the current experimental model.

The manuscript was revised to say:

Our findings support prior work suggesting that an increased oxygen extraction ratio does not necessarily arise from inadequate systemic oxygen delivery (DO□), but rather from heterogeneous microcirculatory blood flow. In this context, microvascular dysfunction resulting from IH can lead to reduce microvascular and irregular perfusion, whereby some regions receive blood flow and oxygen while others are under supplied, resulting in impaired and heterogenous DO□ to peripheral tissues.[12,42,43] As a consequence and documented by our results, peripheral tissues experience persistent regional hypoxia driven IH exposure, rather than sustained reductions in systemic DO□. In the context of chronic obstructive sleep apnea, where such oxygen variations occur nightly, this framework provides a physiological basis for understanding how hypoxic burden and IH may progressively impair microcirculation function and further challenge tissue oxygen availability.

Minor comments

1. There are typos throughout the MS such as incorrect opening/closing of brackets or word order (eg the first sentence starts “Sleep breathing disordered” whereas the accepted condition is “Sleep disordered breathing”). These can easily be rectified by careful proof reading.

We thank the reviewer for pointing out this error and have striven to correct them. We agree that typos and minor grammatical issues were present in the original manuscript. The manuscript has now undergone careful proofreading and editing. We hope the reviewer find the changes improve clarity and readability of the manuscript.

2. Many of the figures are labelled with “Intermediate Hypoxia” rather than “Intermittent Hypoxia”

We thank the reviewer for noting this error, and we have revised the figures to correctly say “Intermittent Hypoxia” and not “Intermediate Hypoxia.”

Overall, this MS presents some interesting results on the behaviour of the microcirculation. It is interesting that the IH protocol induced a sustained level of hypoxia in this model – this would increase the hypoxic burden (HB) overall and a recent systematic review concluded that HB was a better CV risk parameter than traditional parameters such as AHI (Coso et al, 2024). The authors may wish to link into this aspect as they propose microvascular dysregulation in OSA.

We thank the reviewer for this insightful suggestion and agree that the sustained hypoxia observed during the intermittent hypoxia (IH) protocol is best interpreted in the context of hypoxic burden rather than traditional event-based metrics alone. In response to this comment, we have revised the discussion to explicitly frame our results within the emerging concept of hypoxic burden.

Reviewer #2 (Remarks to the Author):

I commend the authors on their detailed and thoughtful work in detecting a potentially harmful ischemia reperfusion cycle that evades systemic compensatory mechanisms in a model of obstructive sleep apnea. Suggested revisions are mostly minor.

Thank you very much for the time and care you devoted to reviewing our manuscript. Your thoughtful comments and constructive suggestions were extremely helpful and have significantly strengthened the clarity, rigor, and overall quality of the work.

We sincerely appreciate your expertise and guidance, which greatly improved the manuscript.

Introduction

1. Line 1 “sleep breathing disordered” did you mean Sleep breathing disorders? Or Disordered sleep breathing?

We thank the reviewer for pointing this typo and have adjusted it accordingly.

2. Line 3, adjust text color to black.

We thank the reviewer for pointing this typo and have adjusted it accordingly.

3. Line 7 try “triggers physiological changes in …”

We thank the reviewer for the important suggestion and have revised the manuscript to reflect the change.

4. End of introduction: You could include systemic compensatory adjustments in your statement of "compensatory microvascular adjustments are insufficient" since you report cardiovascular parameters and blood chemistry.

We thank the reviewer for the suggestion and have adjusted the manuscript accordingly.

Manuscript now reads:

We hypothesize that, in response to the hypoxic burden created by intermittent hypoxia, systemic compensatory adjustments and microvascular adaptations may be insufficient to preserve effective oxygen delivery, resulting in reduced tissue oxygen availability and altered hemodynamics.

Methods

1. Instead of "approved by local animal care committee" state the IACUC approval number.

We thank the reviewer for the suggestion and have added the IACUC approval number for clarity.

Results

1. Changes in Microvascular Resistance: Contains a lot of discussion material. A result's purist would suggest sticking to the facts about microvascular resistance and discussing the "paradox" later. Also:
You state that "despite the reductions in MAP and blood flow, vascular resistance decreased." An alternate explanation that fits with your microvascular narrative is that arteriolar dilation, and decreased vascular resistance, are responsible for the decrease in MAP.

We thank the reviewer for the suggestions and have adjusted the vascular resistance section of the manuscript as follows:

With a minor reduction in MAP and a significant decrease in blood flow, vascular resistance increased over time in both the IH and control groups, across small arterioles (<60 μm), large arterioles (>60 μm), and venules (20–80 μm) (**Figure 4**). As expected, the increase in vascular resistance was more pronounced in the IH group, with the greatest deviation from baseline in larger arterioles followed by the small arterioles and finally the venules. In contrast, the control group exhibited a mild increase in resistance over time.

We'd also like to point out an inconsistency we had with the calculations for vascular resistance. What was originally plotted was Q/MAP, we have since adjusted Figure 4 to properly represent MAP/Q.

2. Calculation for OER is not described in methods or results.

We thank the reviewer for pointing out this error. We revised the methods to include the calculation for OER, the manuscript now reads:

The oxygen extraction ratio (OER), defined as the percentage of O_2 used, was computed as the ratio between VO□ and DO□ (VO_2 / DO_2) at each time point.

Discussion:

1. "at the systemic level … significant reduction in MAPT and initial elevation of HR, indicative of autonomic dysregulation." Dysregulation indicates the cardiovascular system is responding abnormally. The system appears to be responding normally to a mild and sustainable shift to anerobic respiration (as evidenced by IH lactic doubling from BL and holding steady throughout the IH protocol) brought on by decreased DO2. Suggest toning down the paragraph from "overwhelmed" to focus on the observation that the IH CV system is

sufficiently stressed to raise risk for the downstream effects you describe in the next paragraph. The language used in the conclusion conveys the appropriate perspective.

We agree with the reviewer and adjust the paragraph to move away from a dysregulation to more of a natural physiological response due to the oxygen sensing mechanics of carotid bodies and chemoreceptor activation.

At the systemic level, the acute IH challenge elicited a compensatory cardiovascular response, characterized by a reduction in MAP and a transient elevation in HR. Although previous studies have reported hypertension during IH,[23,24] the differences observed here likely reflect several factors, including the lack of experimental control of ventilation, physiological variability inherent to conscious animals, and species-specific reflex mechanisms in Golden Syrian hamsters with robust baroreflex and ventilatory responses.[25] Consistent with this interpretation, the presence of hypocapnia reflects enhanced ventilation and may influence systemic vascular tone, but changes in arterial pressure likely reflect the combined effects of altered resistance and reduced blood flow in the microvasculature..

2. The use of conscious animals is commendable and often missing in oxygen transport studies. However, where we often want to study conscious events (exercise, injury response, etc) and use anesthetized models, here you are studying an unconscious phenomenon using a conscious model. Suggest discussing whether this is a limitation to the study, and if there is a conscious stress component that may affect systemic responses to the IH protocol.

We agree with the reviewer and have incorporated a limitation section talking about this aspect of the study.

The manuscript now includes the following passage:

Limitation

It should be discussed that these observations were made in conscious animals, which allows preservation of intact reflexes and physiological regulation, but also introduces the possibility that conscious stress responses may contribute to systemic hemodynamic changes during IH exposure. While obstructive sleep apnea is inherently an unconscious phenomenon, the use of a conscious model may accentuate ventilatory, cardiovascular, or neurohumoral responses that would otherwise be reduced under anesthesia or sleep. This represents a potential limitation when interpreting systemic outcomes; however, it also strengthens the relevance of the microvascular findings, which reflect integrated responses occurring in the presence of intact neural and vascular control mechanisms. Future studies incorporating controlled ventilation or sleep-state modeling will be valuable for further dissociating hypoxia-driven effects from conscious stress responses.

Reviewer #3 (Remarks to the Author):

COMMSBIO-25-9534
This manuscript details the results from a study investigating the systemic hemodynamic and local microvascular responses to acute intermittent hypoxia in hamsters. The authors argue that this model has relevance to sleep disordered breathing. Overall, the study appears to have been well performed and the results and discussion reflect the underlying data. Making these measures in the un-anaesthetized state is tricky and the authors should be commended for their overall study design. Despite these positive points, I do have some suggestion for improving clarity as well as a suggestion for an additional experimental group.

Thank you for your thoughtful and constructive evaluation of our manuscript. We greatly appreciate your recognition of the study design and the challenges associated with performing these measurements in the un-anaesthetized state. Your suggestions to improve clarity, as well as your recommendation, were insightful and have helped us further improve the manuscript and presentation of the results. We are grateful for the time and care you devoted to this review, which has meaningfully improved the quality and rigor of our manuscript.

Specific comments

A major theme throughout this manuscript is that acute intermittent hypoxia (AIH) causes vasodilation in the microvasculature as a potential compensatory response to help drive increases in blood flow to maintain oxygen delivery. It is argued that under hypoxic conditions (see constraints on this in comments below) these compensatory mechanisms ultimately fail and despite attempts to extract more oxygen hypoxia is essentially associated with microvascular dysfunction, that may have relevance to what is observed clinically with OSA. Yet, in OSA (or in human lab studies where AIH or AIHH is delivered) there is NOT a corresponding drop in blood pressure. Conversely, in this manuscript (and in other rodent papers too), hypoxia causes a significant drop in MAP. Hence, it is not clear to me if the downstream local microvascular dysfunction reported here is simply caused by a reduction in MAP associated with the hypoxia, or if the impaired responses are due to the hypoxia itself. This could be experimentally tested in one of two ways. Either you could infuse phenylephrine during the 30s hypoxic cycles to hold MAP at baseline – as others have done in rodent studies. Or you could experimentally mimic the repetitive drops in MAP without the hypoxia (for example through repeated hemorrhage whilst observing the drop in BP in real-time). Without one of these two experimental manipulations the reader is left to wonder whether the presented microvascular dysfunction is really due to the hypoxia or is simply a result of reduced driving pressure to reduced MAP.

We thank the reviewer for this thoughtful and important comment. We agree that distinguishing between direct effects of hypoxia and secondary consequences of reduced mean arterial pressure (MAP) is critical for interpreting microvascular responses. In response to this concern, we have revised the Discussion to carefully frame systemic cardiovascular changes as reflex-mediated responses involving carotid body and peripheral chemoreceptor activation, rather than as primary drivers of microvascular dysfunction.

Importantly, while intermittent hypoxia in this and other rodent studies is associated with a reduction in MAP, likely reflecting species-specific ventilatory and chemoreflex responses in conscious animals, our data suggest that the observed microvascular behavior cannot be explained solely by reduced driving pressure. In the present study, arteriolar vasodilation occurred despite a fall in MAP, indicating an active myogenic and metabolic response rather than passive pressure-dependent vascular response microcirculation. More importantly, the oxygen tension changes documented during the intermittent hypoxia challenge never reverted during the reoxygenation phase, in fact they were worsen as the cycles of intermittent hypoxia continued during the protocol.

This interpretation is further supported by complementary experiments using chemically induced hypoxia via reducing carrying oxygen capacity, using transfusion of methemoglobin (metHb) saturated RBCs (metRBCs), which reduces blood oxygen-carrying capacity without altering inspired oxygen concentration or blood rheology. In these experiments, metRBCs exchange transfusion similarly produced a reduction in MAP accompanied by arteriolar vasodilation, closely mirroring the microvascular phenotype observed during intermittent hypoxia. The consistency of microvascular responses across both environmental (IH) and exchange transfusion with blood cells unable to transport oxygen supports the conclusion that hypoxic signaling itself, rather than hypotension alone, drives the observed vasodilatory responses.

Cabrales, P., Tsai, A. G., & Intaglietta, M. (2008). Isovolemic exchange transfusion with increasing concentrations of low oxygen affinity hemoglobin solution limits oxygen delivery due to vasoconstriction. American Journal of Physiology-Heart and Circulatory Physiology, 295(5), H2212-H2218. httts://doi.org/10.1152/ajpheart.00751.2008.

J. Martinez, C. Munoz, D. Lucas, et al., " Microvascular Consequences of Methemoglobin Compared to Reduced Hemoglobin in the Microcirculation," Microcirculation 32, no. 6 (2025): e70022, https://doi.org/10.1111/micc.70022.

Moreover, in our previous work using the same dorsal window chamber model under hemorrhagic shock, we have consistently observed that reductions in MAP lead to arteriolar vasoconstriction resulting from myogenic response and not vasodilation. The myogenic response is a blood vessel's intrinsic ability to constrict when pressure rises (due to stretch) and dilate when pressure falls, crucial for regulating blood flow and pressure,

especially in arterioles, protecting tissues from fluctuations, and maintaining autoregulation; in hypotension, the myogenic response causes vasodilation to try and increase flow. Thus, hypoxia and hypotension produce fundamentally different microvascular responses within this experimental framework.

Ref: Liu, Yanping, David R. Harder, and Julian H. Lombard. "Interaction of myogenic mechanisms and hypoxic dilation in rat middle cerebral arteries." American Journal of Physiology-Heart and Circulatory Physiology 283, no. 6 (2002): H2276-H2281.

We agree that experimental approaches such as pharmacological maintenance of MAP (e.g., phenylephrine infusion) or isolated hypotension without hypoxia would provide additional mechanistic insight. While such interventions were beyond the scope of the present study, we now explicitly acknowledge this limitation and outline these strategies as important directions for future investigation.

Overall, we have revised the manuscript to emphasize that the microvascular dysfunction observed during acute intermittent hypoxia likely reflects local oxygen-sensing and chemoreceptor-mediated signaling superimposed on systemic reflex responses, rather than being solely attributable to reduction in arterial blood pressure.

Minor comments / suggestions

1. Do you know the volume of the restraining tube, and have you checked our quickly the air change engulfs the full tube? This may have implications for how long the animal is actually exposed to the hypoxia for

We thank the reviewer for raising this important methodological consideration. The restraining tube used in these experiments has an internal diameter of 5 cm and a length of 15 cm, corresponding to a total internal volume of approximately 295 mL (calculated as a cylinder). The Golden Syrian hamster (~70 g) occupies 80% of the volume; based on body size, leaving an effective gas volume of roughly 60 mL.

Oxygen was delivered directly into the restraining tube at a flow rate of 1 L/min (≈16.7 mL/s). At this flow rate, the residual gas volume within the tube is exchanged in approximately 4 seconds, indicating that the inspired gas composition within the tube rapidly equilibrates with the delivered gas mixture at the onset of each hypoxic or reoxygenation phase. Therefore, the 30-second hypoxic cycles used in this study ensure that animals are exposed to the intended oxygen concentration for the majority of each cycle. We also measure the oxygen concentration in the tube, and after 10 seconds the gas in the tube reached equilibrium with the air supplied into the restraining tube, in addition similar effects were observed during the reoxygenation. Therefore, while the oxygen concentration in the restraining tube might be delayed from the inlet gas, but the time of exposure to hypoxia and reoxygenation remain similar to the gas cycle time.

We have clarified the restraining tube dimensions, effective gas volume, and gas exchange considerations in the Methods section to address this point.

The manuscript now includes the following passage

Given the small residual gas volume within the restraining tube (~60 mL) relative to the gas delivery flow rate of 1 L/min, complete gas exchange occurs within seconds, ensuring rapid onset of hypoxic and reoxygenation exposure during each cycle.

2. Your MAP data is very high – presumably this is a stress response to the tube. Was there any familiarization of the animals with the tube ahead of the experiment? Telemetric studies from other rodents would suggest MAP should be in the range of 90mmHg.

We thank the reviewer for this important comment. All animals underwent prior familiarization with the restraining tube before experimental measurements. Specifically, upon arrival, the same restraining tube used during experiments was placed directly into the animal's cage, allowing animals to freely explore and habituate to it for a minimum of three days prior to any surgical procedures.

During experiments, animals were not head-fixed; however, movement of the dorsal window chamber was restricted to maintain optical alignment for intravital imaging. We acknowledge that this partial restraint differs from fully unrestrained telemetric studies, which typically report lower MAP values (~90 mmHg). In contrast, restrained but conscious preparations, such as dorsal window chamber models, often exhibit higher baseline MAP values due to lack of anesthetic and analgesic. Importantly, baseline MAP values in our study were stable over time (and comparable to laboratory historical data and previously published), and all analyses were performed relative to each animal's own baseline, thereby minimizing the impact of absolute MAP differences on interpretation of hypoxia-induced responses.

3. Is the high PaCO2 normal for a hamster?

We thank the reviewer for raising this point. The arterial PaCO□ values reported in our study (59.2 ± 6.9 and 55.5 ± 5.4 mmHg) are within the physiological range reported for conscious Syrian golden hamsters, as they are fossorial animals and it is comparable to historical data published for hamsters.

In the foundational study by Torres Filho et al. (1996) measured the arterial PaCO□ in awake hamsters breathing room air averaged 55-60 mmHg. Importantly, that study also demonstrated substantial inter-animal variability and noted that PaCO□ values are influenced by conscious state, restraint, and experimental preparation, all of which apply to the present model. Thus, PaCO□ values in the mid-to-high 55 mmHg range fall within the reported physiological range for this specie and preparation.

Baseline PaCO□ values were stable within animals, and all experimental comparisons were performed relative to each animal's own baseline, minimizing the impact of absolute PaCO□ differences on interpretation of hypoxia-induced responses.

4. For the vascular resistance measures, how did you estimate local pressure from MAP?

We thank the reviewer for this comment. Local microvascular pressure requires anesthesia and removal glass cover in the dorsal window, thus pressure in small arterioles was not measured directly in the present study. For relative vascular resistance calculations, we used mean arterial pressure (MAP) as a surrogate for the upstream driving pressure and computed a relative resistance index as:

$$R_{rel} = \frac{MAP/Q}{(MAP/Q)_{baseline}}$$

where $Q$is the microvascular blood flow measured. This approach assumes that changes in MAP are proportional to changes in the upstream pressure supplying the observed microvessels, and because the data are presented normalized to each vessel's baseline, it minimizes the influence of unknown absolute pressure offsets and inter-animal variability. We acknowledge that MAP is an approximation of local perfusion pressure and does not account for vessel specific pressure changes across the arterial tree, or potential regional differences in microvascular pressure. We have clarified this assumption in the limitations section.

We have included the following passage in the manuscript:

Because microvascular pressure was not measured directly, MAP was used as a surrogate for upstream perfusion pressure, and vascular resistance was calculated as MAP/flow and reported as a baseline-normalized, unitless ratio.

5. You are missing a lot of details from the methods. Please add more details about the animals to your methods. For example, how old were they and how many were there? Also, please rationalize your choice of only male hamsters.

We thank the reviewer for this comment and agree that additional animal details improve transparency and reproducibility. The Methods section has been revised to include animal number, sex, age, and group allocation, as well as a clear rationale for the use of male hamsters.

Briefly, male Golden Syrian hamsters were used exclusively in this study to minimize biological variability associated with estrous-cycle–dependent hormonal fluctuations, which are known to influence vascular tone, inflammatory signaling, and microcirculatory regulation in hypoxia and many other conditions. Because a single sex was used consistently across all experimental groups, sex as a biological variable was not directly assessed, and the conclusions of this study are therefore restricted to male physiology.

The revised Methods now specify that animals were divided into an intermittent hypoxia (IH) group (n = 8) and a control group (n = 8). In the IH group, inspired oxygen alternated between 21% (normoxia) and 10% (hypoxia), whereas control animals underwent identical gas switching between two 21% O_2 sources to control for gas delivery, and switching effects (gas flow and sound) without changes in oxygen concentration.

The following passage is now included in the manuscript:

Male Golden Syrian hamsters (Charles River Laboratories, Boston, MA; age 5-6 weeks of age; body weight 55-65 g) were used in this study. Animals were randomly assigned a priory to either an intermittent hypoxia (IH; n = 8) or control (n = 8) group. Only male animals were used to minimize biological variability associated with estrous-cycle–dependent hormonal fluctuations known to affect vascular tone and microcirculatory regulation. Thus, sex as a biological variable was not assessed, and conclusions are therefore limited to male physiology.

6. Why is your PaO2 changing by ~15% in your control group at 30min.

We thank the reviewer for raising this important point. We'd like to remind the reviewer that animals were conscious and partially restrained, and arterial blood gases in this setting can show physiological variability due to changes in ventilation (not experimentally controlled), stress, arousal, and handling associated with repeated sampling. Because PaO_2 reflects the integrated balance between inspired oxygen, alveolar ventilation, and gas exchange, small fluctuations are expected even under nominally constant fraction of inspired oxygen (FiO_2).

Importantly, these changes were not accompanied by a sustained reduction in microvascular oxygenation comparable to the changes observed IH group, and all analyses were performed relative to group baselines to account for inter-animal variability.

7. More details about the time points of the measurements are needed. If I understand correctly, you are continuously cycling gases in the hypoxia group – if this is the case then when in the cycling do you perform your measurements and over what period of time are they made? I assume they are made during a hypoxia cycle, but if so – how long into a hypoxia cycle as the whole cycle lasts only 30s? This may explain the quite high variability in your blood gases and MAP data.

We thank the reviewer for requesting clarification regarding the timing of measurements related to the gas-cycling protocol. We apologize for the lack of clarity in the original Methods and have revised the text accordingly.

Importantly, baseline measurements were obtained before the initiation of any gas cycling. After placement in the imaging setup, animals were allowed to stabilize for 20 minutes. Baseline microvascular parameters, arterial blood gases, and systemic hemodynamics were then recorded while the animal remained in a normal, non-cycling state.

Following baseline acquisition, the intermittent hypoxia (IH) protocol was initiated, during which inspired oxygen was cycled continuously between 21% and 10% O_2 with 30-s intervals. Subsequent measurements at 30 minutes and 60 minutes were obtained while the cycling protocol was ongoing. Data were not collected at a single instant within a given 30-s hypoxic phase; rather, microvascular imaging variables (diameter, RBC velocity/flow, FCD, and hyperspectral oxygenation metrics) were acquired over a multi-minute acquisition window

while cycling continued. Arterial blood gases and MAP/HR were sampled during this same period and therefore represent integrated physiological responses across multiple hypoxia–normoxia cycles, rather than a snapshot from a specific phase of a single cycle.

We agree that sampling during continuous cycling may contribute to variability in systemic measures such as PaO_2, $PaCO_2$, and MAP, particularly in conscious restrained animals. However, this approach was intentionally chosen to characterize the steady-state physiological condition under sustained oscillatory hypoxic burden, rather than transient within-cycle extremes. We have revised the Methods section to explicitly clarify the baseline condition, the timing of measurements relative to gas cycling, and the averaging approach used for data acquisition.

The following passage was added to the manuscript for clarity:

Baseline measurements were obtained under steady normal conditions (21% O_2) following a 20-min stabilization period and prior to initiation of gas cycling. Intermittent hypoxia was then applied (switching from 21% to 10% O_2 every 30 s), and measurements at 30 and 60 min were acquired over various minutes during continuous gas cycling, yielding values integrated across multiple hypoxic–normoxic transitions.

8. In many places you write intermediate hypoxia but this should be intermittent hypoxia.

We thank the reviewer for bringing this to our attention and have revised the manuscript accordingly.

9. Table 1 shows no significant differences in HR but Figure 2 shows increases in HR – which is correct?

We thank the reviewer for bringing this to our attention and have revised the table to reflect its significance to the control.

10. Please thoroughly check your spelling and grammar throughout as there are a number of small mistakes

We thank the reviewer for taking the time to read through our manuscript. We really appreciate it. We have striven to correct all spelling and grammatical errors.

11. Since you do not have any direct "read-out" of autonomic functon(i.e., nerve recordings), I would refrain from making inferences about sympathetic outflow, or autonomic failure, in the paper – as this appears to be largely based on your HR data which could be a response to either sympathetic activation, parasympathetic withdrawal, or simply to maintain cardiac output in the face of reducing stroke volume.

We thank the reviewer for this important clarification and agree that, in the absence of direct measures of autonomic activity (e.g., sympathetic nerve recordings or pharmacological blockade), definitive conclusions regarding sympathetic outflow or autonomic failure cannot be made. We acknowledge that changes in heart rate alone cannot distinguish between sympathetic activation, parasympathetic withdrawal, or compensatory adjustments aimed at maintaining cardiac output in the setting of altered stroke volume.

In response to this comment, we have revised the manuscript to remove inferences regarding autonomic dysfunction or sympathetic dominance. The Discussion has been reframed to describe the observed heart rate changes as nonspecific systemic cardiovascular responses that may reflect integrated reflex mechanisms, including ventilatory influences, baroreflex engagement, and chemoreceptor-mediated signaling, rather than direct evidence of altered autonomic balance.

Manuscript Number: COMMSBIO-25-9534.R2

Title: Intermittent Hypoxia Associated with Sleep Apnea Disrupts Microvascular Hemodynamics and Oxygen Delivery

Dear Editor and Reviewers,

We sincerely thank the Editor and the Reviewers for their careful evaluation of our manuscript and for their detailed and constructive feedback. We appreciate the recognition of the challenges involved in studying this topic in an unanesthetized animal model. We are grateful for all the insightful comments provided by the reviewers as they have improved the manuscript quality, presentation, methodological transparency, and data interpretation.

We have carefully taken into consideration all the comments and suggestions provided and implemented changes in response to each suggestion. We have undertaken revisions to address all comments raised.

Below, we provide point-by-point responses to the Editor's requirements and the Reviewer's comments. All corresponding changes have been incorporated into the revised manuscript and are highlighted in red, while our responses are shown in *blue italics*. Additional clarifications have been incorporated into the Introduction, Methods, Results, Discussion, and the Limitations section.

Thank you,

All authors

Manuscript Number: COMMSBIO-25-9534.R2

EDITOR AND REVIEWERS' COMMENTS:

Response to editorial and reviewers' comments are in *italics in blue*, and changes to the manuscript are shown in red.

Reviewer #1 (Remarks to the Author):

In this revised manuscript the authors have worked through the original comments and provided answers to all the questions raised as well as amending the manuscript where appropriate.

These amendments have improved the clarity of the manuscript and so significantly improved the accuracy and quality, however, there still appears to be one area that seems contradictory.

In the Results>Microvascular Hemodynamics section, the description is of significant vasodilation in both small and large arterioles (Figure 3), however, the following section Results>Changes in Microvascular Resistance reports increases in vascular resistance in both small and large arterioles (Figure 4).

The authors may consider that in Figure 3, the IH group are not actually showing arteriolar vasodilation but just showing a lack of constriction (ie the fall in flow is purely dependent on the MAP falling with no diameter change from baseline)? This may solve the apparent contradiction between these results section.

The overall improvements in the manuscript are welcome and understanding the behaviour of the microcirculation under different conditions is important to our understanding of the cardiovascular consequences of conditions such as OSA.

We thank the reviewer for the thoughtful comments and observations and for recognizing the improvements made to the manuscript. We agree that the interpretation of the diameter changes could reflect either active vasodilation or a relative lack of vasoconstriction in the setting of reduced blood pressure (MAP). To address this potential ambiguity, we have revised the Discussion to acknowledge that the apparent increase in arteriolar diameter during IH may represent a loss of vascular tone rather than active vasodilation to regulate blood flow. This clarification helps reconcile the observed reductions in blood flow with the reported changes in vascular resistance and improves the interpretation of the microvascular hemodynamic responses under intermittent hypoxia.

Text was revised in the discussion to state:

Within this context, our findings suggest that even acute IH may be sufficient to perturb vascular function and oxygen transport. Prior studies examining chronic IH have reported alterations in nitric oxide signaling, increased oxidative stress, and elevated inflammatory mediators, processes known to influence microvascular oxygen delivery.[25,30,31] In our study, even in this acute IH setting microvascular diameters increased while blood flow decreased, suggesting a microvascular remodeling. One potential explanation for this pattern is hypoxic vasodilation, a compensatory response whereby blood vessels dilate in response to reduced oxygen availability to preserve tissue oxygenation.[15,32] However, it is also possible that the apparent increase in diameter reflects a relative lack of vasoconstrictive tone during the IH-induced reduction in MAP, rather than active dilation per se. Regardless of the underlying mechanism, the persistence of reduced microvascular flow, reduced capillary perfusion, and increased vascular resistance despite these diameter changes indicates that such compensatory mechanisms may be insufficient to normalize perfusion under IH conditions.

Manuscript Number: COMMSBIO-25-9534.R2

Reviewer #2 (Remarks to the Author):

Great work! Concerns addressed.

Thank you very much for your thoughtful and constructive review of our manuscript. We truly appreciate the time and effort you devoted to evaluating our work. Your comments and suggestions were extremely helpful and provided important guidance. Thank you again for your contribution and for helping us strengthen the manuscript.

Reviewer #3 (Remarks to the Author):

Thank you for your responses to my suggestions.

I have only one comment left and that is that the limitations should also specifically identify that only male hamsters were used and that responses may differ in female hamsters.

We thank the reviewer for this important comment. We agree that the exclusive use of male hamsters represents a limitation of the study and that the responses observed may differ in female hamsters. We have revised the Limitations section to note that only male hamsters were used and that sex-related differences in responses to estrogen protection and intermittent hypoxia may exist. We also note that future studies including female hamsters will be necessary to determine whether the observed effects are sex dependent.

Limitation

It should be discussed that these observations were made in conscious animals, which allows preservation of intact reflexes and physiological regulation but also introduces the possibility that conscious stress responses may contribute to systemic hemodynamic changes during IH exposure. While obstructive sleep apnea is inherently an unconscious phenomenon, the use of a conscious model may accentuate ventilatory, cardiovascular, or neurohumoral responses that would otherwise be blunted under anesthesia. This represents a potential limitation when interpreting systemic outcomes; however, it also strengthens the relevance of the microvascular findings, which reflect integrated responses occurring in the presence of intact neural and vascular control mechanisms. Additionally, only male hamsters were used in this study, and sex-related differences in vascular regulation, hormonal implications, and hypoxic responses may influence microvascular and systemic adaptations to intermittent hypoxia (particularly 17β-estradiol, which acts as a potent protective agent against hypoxia-induced damage by reducing oxidative stress, inhibiting inflammation, and regulating cellular survival). Therefore, the findings presented here may not fully capture potential sex-dependent physiological responses. Future studies incorporating both sexes and controlled ventilation or sleep-state modeling will be valuable for further dissociating hypoxia-driven effects from conscious stress responses.
